# Beurteilung der Dimensionalität und strukturellen Stabilität der Reflexionsfähigkeit angehender Naturwissenschaftslehrkräfte

**DOI:** 10.1007/s40573-025-00186-7

**Published:** 2025-11-27

**Authors:** Florian Furrer, Christoph Gut, Knut Neumann, Annabel Oehen, Josiane Tardent, Markus Wilhelm

**Affiliations:** 1 Pädagogische Hochschule Zürich, Lagerstraße 2, 8090 Zürich, Schweiz; 2 Pädagogische Hochschule Thurgau, Unterer Schulweg 3, 8280 Kreuzlingen 1, Schweiz; 3 Didaktik der Physik, IPN – Leibniz-Institut für die Pädagogik der Naturwissenschaften und Mathematik, Olshausenstr. 62, 24118 Kiel, Deutschland; 4 Pädagogische Hochschule Luzern, Sentimatt 1, 6003 Luzern, Schweiz

**Keywords:** Reflexionsfähigkeit, Modellierung, Messung, Validität, Reflective Teaching, Modeling, Measuring, Validity

## Abstract

Diese Studie untersucht die Reflexionsfähigkeit – die Fähigkeit, Aspekte eines Unterrichts qualitativ gut reflektieren zu können – von Studierenden für das Lehramt. Die Studie verfolgt einen fachdidaktischen Fokus im Bereich der Naturwissenschaften, da hierzu bislang nur wenige Untersuchungen vorliegen. Die Studierenden führen zwei verschiedene Lektionen zu je 90 min mit unterschiedlichen Experimenten aus Biologie und Chemie, jedoch mit dem identischen Lernziel des naturwissenschaftlichen Experimentierens als Erkenntnismethode, durch und reflektieren diese fachdidaktisch. Es wird ermittelt, ob die Struktur der Reflexionsfähigkeit konsistent erfasst werden kann und inwiefern diese Struktur über mehrere Unterrichtssituationen stabil ist. Des Weiteren wird untersucht, welche Dimensionalität sich empirisch zeigt. Es werden *N* = 61 Studierende im integrierten Fach Naturwissenschaften und Technik der Sekundarstufe I untersucht. Zur Messung wurde ein standardisiertes Instrument zur Erfassung der Reflexionsbreite und Reflexionstiefe entwickelt. Es umfasst 17 Items, welche auf fachdidaktische Aspekte des Unterrichts fokussieren. Die Datengewinnung erfolgte durch semistrukturierte Leitfadeninterviews, welche mittels qualitativer Inhaltsanalyse ausgewertet wurden. Die Ergebnisse deuten auf eine eindimensionale Struktur der Reflexionsfähigkeit hin. Theoretisch fundierte mehrdimensionale Modelle lassen sich in den Daten nicht nachweisen. Diese eindimensionale Struktur ist in beiden Experimentiersituationen vergleichbar identifiziert worden. Die Befunde deuten auf eine strukturelle Stabilität der gemessenen Reflexionsfähigkeit über beide Reflexionsanlässe hin. Die Ergebnisse werden vor dem Hintergrund diskutiert, dass trotz der starken Kontrolle der Erhebung mit der Volatilität solcher Unterrichtssituationen eine große methodische Herausforderung der Messung der Reflexionsfähigkeit einhergeht. Es stellt sich dabei die Frage, wie viele Unterrichtssituationen für eine valide und reliable Einschätzung der fachdidaktischen Reflexionsfähigkeit notwendig wären.

**Zusatzmaterial online:**

Zusätzliche Informationen sind in der Online-Version dieses Artikels (10.1007/s40573-025-00186-7) enthalten.

## Abstract (en)

This study examines the reflective ability—the ability to reflect with quality on a lesson’s aspects—of pre-service students teachers. The present study pursues a subject-specific focus in the natural sciences, as there have been only a few such studies to this date. To this end, the students conduct two different 90 min lessons, each featuring distinct experiments from the fields of biology and chemistry, but with the identical learning objective of scientific experimentation as a method of acquiring knowledge. Subsequent to the lesson, the students engage in subject-specific reflection on the lessons. It is determined whether the structure of the subject-specific reflection ability can be consistently recorded and to what extent this structure is stable over multiple teaching situations. In addition, the study examines which dimensionality is empirically identifiable. A total of 61 pre-service science teachers in the integrative subject of science and technology at lower secondary level were examined. A standardized instrument was developed to measure the breadth and depth of reflection. It comprises 17 items that focus on subject-specific aspects of reflection. Data was collected through semi-structured guided interviews, which were analyzed using qualitative content analysis. The analysis of the structure suggests a one-dimensional construct for the ability to engage in subject-specific reflection. Multidimensional models based in theory are not supported by the data. This one-dimensional structure can be identified in a comparable manner in both experimental situations. The findings indicate a structural stability of the measured ability across both reflection occasions. The results are discussed against the background that, despite the survey’s rigorous control, the volatility of such teaching situations poses a major methodological challenge for measuring reflection. This raises the question of how many teaching situations would be necessary for a valid and reliable assessment of the ability to engage in subject-specific reflection.

## Einleitung

Es gehört zum Wesen des Menschen, dass er seine Handlungen reflektiert. Der Erwerb einer professionellen Fähigkeit des Reflektierens, die dispositionale Eigenschaften aufweist, erfordert jedoch die Sozialisierung mit den spezifischen Formen, wie in einer Domäne, beispielsweise in Bezug auf die Tätigkeit als Lehrkraft oder auf eine bestimmte Fachdisziplin (Häcker 2019), reflektiert wird bzw. werden sollte. In der Domäne des Lehrberufes gilt die Fähigkeit des gezielten Nachdenkens über den eigenen Unterricht als ein zentrales Ziel der Professionalisierung. Hierbei wird das Handeln kritisch mit Theorie in Bezug gesetzt bzw. situatives Handeln mit dem eigenen Wissen verknüpft (von Aufschnaiter et al. 2019). Von dieser Fähigkeit wird angenommen, dass sie ohne systematische Schulung nicht erworben werden kann (Wyss 2013). Qualitätsvolles Reflektieren soll nicht nur das Professionswissen fördern, sondern korreliert tatsächlich auch mit der Qualität des eigenen Unterrichts (Baumert und Kunter 2013; Cojorn und Sonsupap 2022; Loughran 2002).

Aufgrund des Stellenwerts, welcher der Fähigkeit zum qualitätsvollen Reflektieren im aktuellen Forschungsdiskurs zukommt, rücken Fragen der Modellierung und Messung einer solchen Fähigkeit von Lehrkräften in den Fokus der Unterrichtsforschung. Dabei zeigen sich derzeit noch verschiedene Schwierigkeiten: Einerseits findet sich in der Literatur keine einheitliche, allgemein akzeptierte Definition, wie die Reflexionsfähigkeit modelliert werden soll (Kempin et al. 2020, Wyss 2013). Andererseits wurden bisher diverse Instrumente für die Messung einer solchen Fähigkeit entwickelt, die sich in Bezug auf den Unterricht, der reflektiert werden soll (Stimulus), auf die vorgegebenen Kriterien, nach welchen reflektiert werden soll (Aufforderung), sowie auf die Kategorien, nach welchen die evozierten Reflexionen beurteilt werden (Bewertungsraster), unterscheiden (Wyss 2013; Mientus et al. 2023). Darüber hinaus kommen bei der Messung unterschiedliche Ansätze zur Anwendung: Es gibt Messinstrumente, die mit verschiedenen Stimuli das zu messende Konstrukt erfassen und dadurch implizit eine Messwiederholung beinhalten, und es gibt solche, die mit einem Stimulus das ganze Konstrukt erheben und daher keine Wiederholung der Messung beabsichtigen.

Ob eine Messung eine Messwiederholung mit einschließt, ist im Rahmen einer Validierung der Messung deshalb eine relevante Frage, weil ohne empirische Überprüfung nicht davon ausgegangen werden darf, dass sich die Performanz einer Testperson beim Reflektieren von Unterricht über verschiedene Anforderungssituationen (Stimuli und Aufforderungen) und Messzeitpunkte hinweg als stabil genug erweist, um eine strukturell stabile Skalierung einer entsprechenden latenten Reflexionsfähigkeit zu erreichen. Diese Frage stellt sich im Besonderen, wenn der Stimulus „realer“ Unterricht ist. Im Gegensatz zu beispielsweise Unterrichtsvignetten ist bei „realem“ Unterricht eine hohe, nicht kontrollierbare Situationsabhängigkeit gegeben, die die Volatilität der Performanz bei der Reflexion zusätzlich erhöhen kann.

Der Wunsch, die Reflexionsfähigkeit einer Lehrkraft anhand der Performanz bei einem einmaligen Reflektieren eines „realen“ Unterrichts erfassen zu können, rührt u. a. von dessen hohen ökologischen Validität in Bezug auf Leistungssituationen im Professionalisierungskontext des Lehrerseminars und der Schulpraxis her. Obwohl der Ansatz auch in den naturwissenschaftlichen Didaktiken sehr oft gewählt wird, wurde der Nachweis bisher nicht erbracht, dass solche Erhebungen die Skalierung eines, über verschiedene Messungen hinweg, strukturell stabilen Konstrukts ermöglichen. Dieser Forschungslücke widmet sich die hier vorgestellte Studie, bei der 61 Studierende zwei Mal einen selbst geplanten und erteilten Unterricht an einer Klasse im Rahmen eines semistrukturierten Interviews reflektiert haben. Die kodierten und skalierten Interviews wurden genutzt, um zu überprüfen, inwiefern dieser Messansatz eine strukturell stabile Messung erlaubt.

## Theoretischer Rahmen

Das Handeln von Lehrkräften gliedert sich in die drei Phasen des Lehrzyklus: Planen, Unterrichten und Reflektieren. Der dritten Phase – dem Reflektieren – kommt dabei die Rolle zu, aus den ersten beiden Phasen Erkenntnisse für einen weiteren Lehrzyklus bzw. für weitere Lehrzyklen zu gewinnen. Dabei sollen Entscheidungen in Ruhe und mit der notwendigen Distanz durchdacht und analysiert sowie Rückschlüsse für zukünftiges unterrichtliches Handeln gezogen werden (Meyer-Siever 2019). Eine fundierte Unterrichtsreflexion sollte daher trotz retrospektiven Grundcharakters auf prospektive Ziele ausgerichtet sein.

Um die Reflexionsfähigkeit von Lehrkräften als psychometrisches Konstrukt zu messen, braucht es einerseits einen Stimulus, der Inhalte der ersten beiden Phasen eines Lehrzyklus vorgibt, und andererseits eine Aufforderung, die eine Reaktion in Form einer Unterrichtsreflexion erforderlich macht. Stimuli, Aufforderungen und Raster für die Bewertung der Reaktion sind konstitutive Elemente eines Messinstruments (Ruiz-Primo und Shavelson 1996). Sie sind gleichzeitig auch Ausdruck der dahinterliegenden Modellierung des zu messenden Konstrukts. Für die Validität einer Messung der Reflexionsfähigkeit ist es nun entscheidend, inwieweit diese Modellierung theoriebasiert sowohl auf die Auswahl, Gestaltung und gegebenenfalls vorhandene Variation des Stimulus und der Aufforderung als auch auf die Bewertung der Reaktion in Form einer Unterrichtsreflexion eingeht (Schreiber und Gut 2022). In den folgenden Abschnitten sollen aus dieser Perspektive bestehende Modellierungsansätze und Messinstrumente der Reflexionsfähigkeit diskutiert und auf bestimmte Fragen der Validität von Messungen eingegangen werden.

### Reflexionsfähigkeit modellieren – Reflexionstiefe und Reflexionsbreite

Mit der Modellierung eines Kompetenzkonstrukts wie der Reflexionsfähigkeit sind im Allgemeinen drei Aufgaben verbunden: die Abgrenzung des Konstrukts in seinem Umfang, die innere Differenzierung des Konstrukts durch die Annahme von Teilfähigkeiten sowie die Festlegung, wie die Güte oder Entwicklung der Fähigkeit (Progression) bzw. der Teilfähigkeiten erfasst werden sollen (vgl. Gut und Mayer 2018). Mit der Modellierung als Antwort auf die drei Aufgaben werden im Idealfall explizite a priori-Setzungen gemacht, die in die Entwicklung eines Messinstruments, sprich die Auswahl der Stimuli, die Formulierung der Aufforderungen sowie die Setzung der Beurteilungsraster, eingehen und die im Rahmen einer Validierung von Messungen u. a. anhand empirischer Daten einer „Überprüfung“ unterzogen werden (Schreiber und Gut 2022). In Bezug auf die Messung von Teilfähigkeiten und Progressionen kommen dabei grundsätzlich zwei Ansätze zur Anwendung: Beim Ansatz „Modellierung durch Variation der Anforderungssituation“ werden für verschiedene Teilfähigkeiten bzw. Facetten der Fähigkeit oder für verschiedene Progressionsstufen (Niveaus) separate Aufgaben (Items) mit entsprechend unterschiedlich gearteten bzw. unterschiedlich anspruchsvollen Stimuli und Aufforderungen entwickelt. Eine Messung besteht darin, dass die Testperson ein Set solcher Aufgaben (Items) löst (vgl. z. B. Wöhlke und Höttecke 2022). Beim Ansatz „Modellierung durch Differenzierung bei der Beurteilung“ erhalten Testpersonen einen Stimulus, wobei die Differenzierung von Teilfähigkeiten und die Beurteilung der Progressionsstufe mit der Beurteilung der Reaktion anhand eines Beurteilungsrasters erfolgen (z. B. Wyss 2013). Die Wahl des Ansatzes hat auf die Validierung von Messungen insofern einen Einfluss, als dass nicht jeder Ansatz mit jeder Messtheorie (klassische oder probabilistische) kompatibel ist. Auch erfordert die „Überprüfung“ der a priori-Setzungen in Bezug auf die Progression andere statistische Mittel.

Im Fall der Reflexionsfähigkeit von Lehrkräften besteht derzeit ein großes Forschungsinteresse an der Modellierung gemäß dem zweiten Ansatz. Dabei wird von der impliziten Annahme ausgegangen, dass sich eine professionelle Reflexionsfähigkeit dadurch auszeichnet, dass einerseits das eigene (oder fremdes) Handeln breit, sprich anhand zahlreicher relevanter didaktischer und pädagogischer Aspekte, und andererseits jeder Aspekt fundiert und tiefgründig reflektiert wird (Riel 2022). Im ersteren Fall spricht man von der Breite der Reflexion, im letzteren von deren Tiefe. Obwohl beide „Dimensionen“ als relevant erachtet werden, gibt es bisher nur wenige Untersuchungen, die die Beurteilungen beider Dimensionen kombinieren (Riel 2022).

Die meisten Instrumente, die die Reflexionstiefe erfassen, orientieren sich entweder am Modellierungsansatz von Plöger et al. (2015) oder am Ansatz von Hatton und Smith (1995). Beide Autorenteams beschreiben die Reflexionstiefe als a priori angenommenes, hierarchisch konzipiertes Stufenmodell. Während im Ansatz von Plöger et al. (2015) die analytische Form der Reflexion im Vordergrund steht (*Beschreibung *von diskutablen Unterrichtssituationen, deren *Bewertung, *Nennung von *Alternativen *zu und *Konsequenzen* der genannten Situationen), baut der Ansatz von Hatton und Smith (1995) auf einer holistischen Beschreibung der Unterrichtsreflexion als Diskurstätigkeit auf (*Descriptive Writing, Descriptive Reflection, Dialogic Reflection, Critical Reflection*). Mit der Stufe *Critical Reflection* beziehen Hatton und Smith zudem die Überlegung mit ein, dass Unterricht durch gesellschaftliche, politische und historische Umstände bedingt wird, die Plöger et al. explizit nicht modellieren. Anwendungen und Weiterentwicklungen des Ansatzes von Plöger et al. (2015) finden sich z. B. bei Windt und Lenske (2016), Nowak et al. (2019), Nowak (2023) oder bei Knott et al. (2023). Kempin et al. (2018) weiten die Reflexionstiefe auf sieben Stufen aus, indem sie außer bei der Beschreibung jeweils mittels einer Begründungsstufe eine Differenzierung vornehmen. Der Ansatz gemäß Hatton und Smith (1995) findet u. a. bei Abels (2011) und Grünbauer et al. (2023) Verwendung. Da an der hierarchischen Auffassung solcher Stufenmodelle Kritik geübt wurde, wird inzwischen vorgeschlagen, die Stufen als unabhängig voneinander abzudeckende Bereiche zu verstehen (Riel 2022).

Die bestehende Literatur zu Modellierungen der Reflexionsbreite erscheint heterogen. Dennoch sind zwei übliche theoretische Fundierungen der Modellierung erkennbar (Plöger und Scholl 2014). Zum einen werden die Aspekte der Reflexionsbreite mit Merkmalen der Unterrichtsqualität, insbesondere mit der Weiterentwicklung der generischen Basisdimensionen, in Bezug gesetzt und begründet (Kunter et al. 2011; Steffensky und Neuhaus 2018; Szogs et al. 2019). Den verwendeten Begrifflichkeiten liegen jedoch oft unterschiedliche Verständnisse zugrunde und müssten disziplinspezifisch ausgedeutet werden (Heinitz und Nehring 2020; Steffensky und Neuhaus 2018). Besonders groß sind die Unterschiede, wenn die Aspekte der Reflexionsbreite auf Operationalisierungen der Tiefenstruktur basieren (Steffensky und Neuhaus 2018). Zum anderen orientieren sich Aspekte der Reflexionsbreite an der als notwendig erachteten Wissensbasis der Lehrkraft und an der von Shulman (1986) vorgeschlagenen Unterscheidung von pädagogischem, fachlichem und fachdidaktischem Wissen (Nowak et al. 2019), wobei die drei Wissensbasen oft zusätzlich und unterschiedlich ausdifferenziert werden (vgl. Davis 2006; Kobl 2021). Insbesondere wird fachdidaktisches Wissen in gewissen Konzeptionen als eigenständige Wissensform betrachtet und in anderen Konzeptionen in das Fachwissen integriert (Gess-Newsome 2002). Fachdidaktisches Wissen dient der Vermittlung fachlicher Inhalte und kann daher nicht ohne Fachwissen existieren. Die beiden stehen deshalb in einem engen Zusammenhang. Das fachdidaktische Wissen ist jedoch in beiden Fällen nicht mit dem Fachwissen deckungsgleich (Kunter et al. 2011, Gramzow et al. 2013).

Bestehende Modellierungen lassen sich nicht immer eindeutig einem dieser beiden Ansätze zuordnen (vgl. Schaal et al. 2023). Eine Ausdifferenzierung erfolgt meist aufgrund des Forschungsinteresses. Dies führt jedoch analog zur Reflexionstiefe dazu, dass Modelle und Erkenntnisse zur Reflexionsbreite über Studien hinweg schlecht verglichen werden können (Riel 2022). Während die Reflexionstiefe ausschließlich für die Progressionsmodellierung der Reflexionsfähigkeit genutzt wird, wird die Reflexionsbreite sowohl zur inneren Differenzierung der Fähigkeit als auch zur Beurteilung deren Ausprägung herangezogen. Im letzteren Fall steht die Annahme im Vordergrund (a priori-Setzung), dass eine quantitativ umfassendere Abdeckung der Aspekte der Reflexionsbreite eine qualitativ höherwertigere Reflexion kennzeichnet, da so der Unterricht komplexer analysiert wird (Riel 2022; Thede et al. 2022). Im ersteren Fall wird davon ausgegangen (a priori-Setzung), dass eine Lehrkraft bei der Reflexion über unterschiedliche Aspekte des Unterrichts auf unterschiedliche Basen des Professionswissens zurückgreift und deshalb Strukturen des Wissens sich teilweise in der Struktur der Unterrichtsreflexion abbilden.

### Reflexionsfähigkeit messen – das Problem der Volatilität

Das psychometrische Grundproblem jeder Kompetenzmessung besteht darin, dass aus der Beurteilung der Performanz einer Testperson in einer oder wenigen isolierten Anforderungssituationen in gerechtfertigter Weise auf eine latente Fähigkeit dieser Person geschlossen werden soll, die deren Performanz in weiteren und anderen Anforderungssituationen reliabel voraussagt. Der Schluss erfordert im Rahmen der Validierung der Kompetenzmessung eine Rechtfertigung in Form einer Validierungsargumentation (vgl. Schreiber und Gut 2022). Das stärkste, in solchen Situationen jeweils vorgebrachte, notwendige, aber nicht hinreichende Argument besteht darin, aufzuzeigen, dass die Messung der latenten Fähigkeit aus einer psychometrisch akzeptablen, sprich reliablen, Skalierung hervorgeht.

Zum psychometrischen Grundproblem gesellt sich das empirische Problem, dass sich die Performanz einer Testperson in vergleichbaren, verschiedenen Anforderungssituationen und/oder zu verschiedenen Testzeitpunkten in der Regel nicht als stabil, sondern als volatil erweist (Brennan 1996; Shavelson et al. 1999). Diese Volatilität wird sogar bei hoch standardisierten Tests unterschiedlichster fachlicher Gattungen festgestellt (Brennan 1996). Man darf also gerade bei komplexen Anforderungen, wie sie bei der Reflexion eines Unterrichts gegeben sind, nicht per se von einer vernachlässigbaren Volatilität ausgehen (vgl. u. a. Hild et al. 2018).

Die Volatilität der Performanz kann messtheoretisch unterschiedlich interpretiert werden. Sofern im Rahmen einer Generalisierbarkeitsstudie empirisch nachgewiesen wird, dass sich verschiedene Messungen zu einer Gesamtmessung verallgemeinern lassen, ist die Volatilität Ausdruck der Ungenauigkeit der Einzelmessung. Auf die Messung der Reflexionsfähigkeit übertragen heißt das z. B., dass für eine belastbare, reliable Aussage zur Reflexionsfähigkeit einer Lehrkraft desto mehr Beurteilungen von Unterrichtsreflexionen benötigt werden, je größer die Volatilität ist. Ist die Generalisierbarkeit hingegen empirisch nicht zu belegen, ist die Volatilität Ausdruck eines mangelhaften Transfers zwischen verschiedenen Anforderungssituationen und somit der fehlenden Bedingung dafür, dass die Performanz als latente Fähigkeit interpretiert werden darf (vgl. Schreiber und Gut 2022).

Das Problem der Volatilität erfordert eine entsprechende Validierung der Kompetenzmessung, die unter Einbezug von Messwiederholungen die Generalisierbarkeit der Messung, sprich einer Art Retest-Reliabilität, überprüft. Eine hierzu notwendige, jedoch nicht hinreichende Validierungsargumentation besteht darin, aufzuzeigen, dass die Skalierung des zu messenden Konstrukts bei verschiedenen Messungen mit variierenden Anforderungssituationen und zu verschiedenen Zeitpunkten strukturell stabil ist. Auf die Messung der Reflexionsfähigkeit übertragen bedeutet diese Anforderung, die im Weiteren „strukturelle Stabilität“ genannt wird, dass sich die Reflexionsfähigkeit als latentes Fähigkeitskonstrukt unabhängig von den konkreten Unterrichtssituationen, die reflektiert werden, immer auf dieselbe Art skalieren lässt und aus der Skalierung immer dieselbe innere Differenzierung von Teilfähigkeiten hervorgeht.

Aufgrund theoretischer Überlegungen erscheint die Anforderung der strukturellen Stabilität an die Messung einer Reflexionsfähigkeit nicht ohne einschränkende Auflagen an die Modellierung des Konstrukts und die Gestaltung des Messinstruments erreicht werden zu können. Die Volatilität der Performanz beim Reflektieren ist nicht nur die Folge eines grundsätzlich volatilen menschlichen Problemlöseverhaltens, sondern sie spiegelt auch die Variabilität in den Anforderungssituationen (Stimulus und Aufforderung) wider, die bei Messwiederholungen mit anderen Stimuli in der Natur der Sache liegt. So ist beispielsweise die Reflexion bei hochstandardisierten Unterrichtsvignetten als Stimuli im Vergleich zur Reflexion der eigenen Unterrichtslektion als deutlich weniger volatil anzunehmen. Textvignetten wie auch Videovignetten unterliegen jedoch der Limitierung, dass eine Unterrichtssituation nur bedingt authentisch abgebildet werden kann (Billion-Kramer et al. 2023; Neuweg 2015). Zusätzlich wird Unterricht anders reflektiert, wenn es nicht der eigene ist (Wyss 2013). Obschon Vignetten auch als nah an der Unterrichtssituation beschrieben werden (Billion-Kramer et al. 2023), liegt der Vorteil dieses Vorgehens darin, dass es sich noch näher an der tatsächlichen Praxis der Lehrkräftebildung befindet, sprich ökologisch valider ist. Darüber hinaus ist anzunehmen, dass inhaltsoffene Aufforderungen zu mehr Volatilität beim Reflexionsverhalten führen, als wenn die Aufmerksamkeit der Lehrkraft gelenkt wird.

Auch die Breite des modellierten Konstrukts könnte die Volatilität der gemessenen Performanz beeinflussen. So ist aus der Unterrichtsforschung bekannt (vgl. Praetorius et al. 2014), dass die professionelle Beurteilung von Unterricht einer Lehrkraft in Bezug auf das allgemeindidaktisch-pädagogische Merkmal *Klassenführung* über verschiedene Lektionen hinweg deutlich weniger volatil ausfällt als in Bezug auf das fachdidaktische Merkmal *kognitive Aktivierung*, dessen Ausgestaltung auch vom Fachkontext und vom Lernziel der einzelnen Lektion abhängt. Es ist plausibel anzunehmen, dass es in Bezug auf verschiedene Aspekte des Unterrichts analoge Unterschiede der Volatilität auch bei der Unterrichtsreflexion durch Lehrkräfte gibt. Für die Reflexion von fachkontext- und lernzielsensitiven Aspekten des Unterrichts wie beispielsweise dem Umgang mit Schüler*innenvorstellungen greift die reflektierende Lehrkraft auf entsprechendes fachinhaltliches und fachdidaktisches Wissen zurück. Ändern sich bei einer Wiederholung der Messung der Fachkontext oder das Lernziel des Stimulus (zu reflektierender Unterricht), ändern sich auch die Wissensvoraussetzungen für die Lehrkraft. Die Lernziel- und Fachkontextabhängigkeit zeigt sich letztlich auch darin, dass für die Kodierung ausgeprägt fachdidaktischer Aspekte der Breite die Kodierschemata situativ auf das Lernziel und den Fachkontext des Unterrichts adaptiert werden müssen (Dorfner et al. 2017).

Das Problem der Volatilität ist nicht für beide Modellierungsansätze gleich virulent. Beim Ansatz „Modellierung durch Variation der Anforderungssituation“ enthält die Messung bereits Messwiederholungen, da mit jedem Item eine zusätzliche Messung gemacht wird. Der Ansatz, nur durch die Beurteilung zu differenzieren, kommt hingegen dann zur Anwendung, wenn eine Kompetenz anhand einer einzelnen Anforderungssituation gemessen werden soll. In Studien, in welchen die Reflexionsfähigkeit von Lehrkräften gemessen wird, kommt dieser zweite Ansatz aus pragmatischen oder inhaltlichen Gründen häufig zum Zug (z. B. soll der eigene Unterricht reflektiert werden). Der Fokus der Modellierung liegt dann auf der Differenzierung von Reflexionsbreite und/oder Reflexionstiefe.

## Forschungsfragen

Die Forschung zur Möglichkeit, die Reflexionsfähigkeit von Lehrkräften in den Naturwissenschaften valide zu messen, ist noch wenig ausgebaut und konsolidiert. Dies betrifft sowohl die Modellierung der Reflexionsfähigkeit als psychometrisches Konstrukt als auch die Entwicklung und Validierung entsprechender Messinstrumente. Etabliert hat sich die Sichtweise, dass die Fähigkeit, Unterricht zu reflektieren, sowohl in der inhaltlichen Breite als auch in der Tiefe beurteilt werden sollte, wobei die inhaltliche Breite allgemeindidaktisch-pädagogische und fachdidaktische Aspekte des Unterrichts betreffen kann. Bestehende Modellierungen und entsprechende Messinstrumente sind jedoch in Bezug auf beide „Dimensionen“ divers.

Breite und Tiefe einer Reflexion bei der Messung zu kombinieren, ist grundsätzlich mit beiden Messansätzen – „Modellierung durch Variation der Anforderungssituation“ und „Modellierung durch Differenzierung bei der Beurteilung“ – umsetzbar. Der einfachste und am häufigsten gewählte Ansatz besteht jedoch darin, Testpersonen singulär einen selbst erlebten oder beobachteten Unterricht reflektieren zu lassen und deren Reflexion zu beurteilen. Dieser Ansatz verspricht aufgrund der hinsichtlich der Professionalisierungskontexte und Leistungssituationen im Referendariat und später im Schulfeld „authentischen“ Anforderungssituation eine hohe ökologische Validität. Im Bereich des Reflektierens von Naturwissenschaftsunterricht wurde jedoch bisher noch nicht gezeigt, dass dieser Ansatz eine strukturell stabile Skalierung der Reflexionsfähigkeit ermöglicht. In der vorliegenden Studie wird deshalb untersucht, inwiefern die Reflexionsfähigkeit von Studierenden für das Lehramt strukturell stabil erfasst werden kann. Die Untersuchung der strukturellen Stabilität wird aufgrund des begrenzten Artikelumfangs nur auf die Reflexionsbreite bezogen. Dabei werden die Forschungsfragen F1 und F2 beantwortet.

### Forschungsfrage F1:

Inwiefern kann die Reflexionsfähigkeit in Bezug auf die Vermittlung eines kontrollierten Lernziels über zwei Unterrichtseinheiten mit derselben Klasse strukturell stabil erfasst werden?

### Forschungsfrage F2:

Sofern die Reflexionsfähigkeit strukturell stabil erfasst werden kann, welche inhaltlich valide Dimensionalität weist die Reflexionsfähigkeit auf?

## Methode

### Erhebungsdesign

**Abb. 1:**
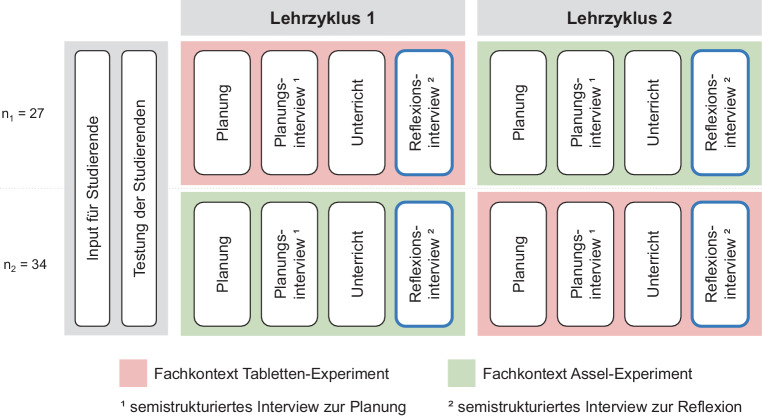
Erhebungsdesign PURPUR

Die Beantwortung der Forschungsfragen erfolgt anhand von Daten, die im Rahmen des übergeordneten Projekts PURPUR erhoben wurden. In diesem Projekt wird die Qualität der Handlungen von Studierenden im vollständigen Lehrzyklus Planen, Unterrichten und Reflektieren erfasst und verglichen. Der Fokus des Messinstruments liegt hierbei auf dem Anteil der Reflexionsfähigkeit, welcher auf Aspekte der Reflexionsbreite mit primär fachdidaktischem Bezug fokussiert (beispielsweise der Umgang mit Schüler*innen-Vorstellungen). Im Folgenden wird auf das Erhebungsdesign des Projekts PURPUR nur so weit eingegangen, wie es für diese Teilstudie von Relevanz ist. Für die Datenerhebung haben Studierende für das Lehramt auf der Sekundarstufe I in einwöchigem Abstand zwei Doppellektionen (2 × 90 min) geplant, durchgeführt und reflektiert. Unmittelbar nach den Lektionen wurde mit den Studierenden jeweils ein semistrukturiertes Reflexionsinterview von einer im Projekt mitarbeitenden Person durchgeführt. Die Teilstudie bezieht sich auf diese Kodierungen und Analysen. Die entsprechenden Erhebungsteile sind in Abb. 1 blau konturiert. Für die Vergleichbarkeit der Erhebungen der Reflexionsfähigkeit erhielten die Studierenden die Vorgabe, in beiden Doppellektionen am gleichen Lernziel (Experimentieren als Erkenntnismethode) zu arbeiten und hierfür mit gegebenem standardisiertem Experimentiermaterial Instruktionen mit Schüler*innen-Experimenten vorzubereiten: einmal mit einem biologischen Fachkontext (Asselexperiment: Untersuchung der Aufenthaltswahrscheinlichkeit in Abhängigkeit von der Helligkeit und Feuchtigkeit; vgl. Shavelson et al. 1991) und einmal mit einem chemischen Fachkontext (Tablettenexperiment: Untersuchung der Lösegeschwindigkeit in Abhängigkeit von der Temperatur und des Volumens des Wassers; vgl. Harmon 1997). Die Wahl der Fachkontexte begründet sich dadurch, dass die Schüler*innen für die Bearbeitung der Experimente kaum curriculares Vorwissen mitbringen müssen und der Unterricht deshalb mit allen Klassen ähnlich gestaltet werden kann. Die Studierenden erhielten vor der Erhebung neben standardisierten Informationen zur Studie im Rahmen einer Repetitionsveranstaltung einen Input zum Experimentieren als Erkenntnismethode. Sie wurden zudem angewiesen, für die Planung der Doppellektionen keine Unterstützung von Mentor*innen und Kolleg*innen anzunehmen. Im Vorfeld wurden das Fachwissen und das fachdidaktische Wissen der Studierenden zu den beiden Fachkontexten erhoben.

### Stichprobe

Bei den Studienteilnehmenden handelte es sich um eine Gelegenheitsstichprobe von Studierenden der Sekundarstufe I im 6. und 7. Semester von drei Pädagogischen Hochschulen in der Schweiz. Die Studierenden werden für das Unterrichten der drei Fächer Biologie, Chemie und Physik im Rahmen eines integrierten Naturwissenschaftsfachs ausgebildet. Von den 85 Studierenden wurden 24 (= 28 %) wegen Unvollständigkeit des Datenmaterials (Abbruch, COVID-Schulschließung) oder zwecks Schulung der Kodierer*innen von der Analyse ausgeschlossen. Von den 61 in die Auswertung einbezogenen Studierenden waren 28 männlich und 33 weiblich. Die unterrichteten Schüler*innen waren zwischen 12 und 15 Jahre alt.

Die Abfolge der Fachkontexte der Doppellektionen wurde möglichst gleichmäßig auf die Studierenden aufgeteilt (Start mit „Asseln“ bei 34 Studierenden und mit „Tabletten“ bei 27 Studierenden). Für die Erfassung der Reflexionsfähigkeit wurden direkt im Anschluss an den Unterricht vor Ort semistrukturierte Leitfadeninterviews durchgeführt. Die Tonaufnahmen der Gespräche wurden manuell transkribiert (Dauer: *M* *=* *25* *min, SD* *=* *6* *min*).

### Ansatz dieser Studie

In der vorliegenden Studie wird eine Erhebung von Reflexionsgesprächen (mündliche Reflexionsanlässe) zu dem von den Studierenden selbst abgehaltenen Unterricht mittels semistrukturierter Leitfadeninterviews umgesetzt. Das verwendete Messinstrument orientiert sich daher an einem inhaltlich geschlossenen Format und legt den Fokus auf die Reflexion fachdidaktischer Aspekte. Das Verständnis der Reflexionstiefe orientiert sich am Ansatz von Plöger et al. (2015) und entwickelt die Modellierung von Nowak et al. (2019) weiter. Zudem wurde die Auffassung übernommen, dass es sich bei der Reflexionstiefe um voneinander unabhängige Bereiche handelt (siehe theoretischer Rahmen). Die Einschätzung der Reflexionsqualität erfolgt durch die Kodierenden.

### Messung der Reflexionsfähigkeit mit fachdidaktischem Fokus

**Abb. 2:**
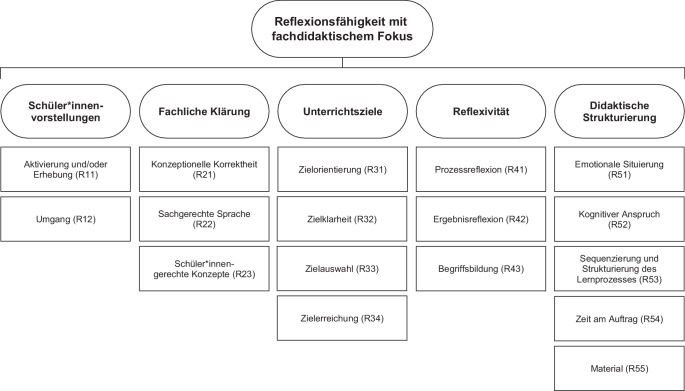
Messinstrument PURPUR‑R zur Messung des Konstrukts der Reflexionsfähigkeit mit insgesamt 17 Items der Reflexionsbreite mit fachdidaktischem Fokus. (Adaption nach Tardent 2020)

Verschiedene Arbeiten befassen sich mit der Operationalisierung von Tiefenstrukturmerkmalen für den naturwissenschaftlichen Unterricht (z. B. Neuhaus 2007). Im Projekt KUBeX wurde eine, auf dem Modell der didaktischen Rekonstruktion (Kattmann et al., 1997) gestützte Operationalisierung auf Unterricht mit dem Lernziel experimentelles Handeln angewandt (Tardent 2020; Weitzel und Blank 2019). Ein entsprechendes Messinstrument mit 19 Items, geordnet in fünf theoretisch hergeleitete Kategorien, wurde für die Erhebung der Planungsfähigkeit von Studierenden erfolgreich strukturell validiert (Tardent 2020). Auf der Basis des KUBeX-Messinstruments wurde im Rahmen des Forschungsprojekts PURPUR ein auf den Unterricht zum Experimentieren adaptiertes Messinstrument für die Reflexionsfähigkeit entwickelt. Das Instrument, das im Weiteren mit PURPUR‑R bezeichnet wird, umfasst 17 Items der Reflexionsbreite, die als gleichwertige Items einer Skala (Reflexionsfähigkeit mit fachdidaktischem Fokus) aufgefasst werden (Abb. 2). Detaillierte Beschreibungen sind im Kodiermanual (Onlinematerial D) ersichtlich.

Die Reflexionstiefe wurde in PURPUR‑R analog zur Modellierung von Nowak et al. (2019) für jedes Item der Breite separat erfasst und als ordinale Progression im Sinne eines *Partial Credit* interpretiert.

### Kodierung

**Abb. 3:**
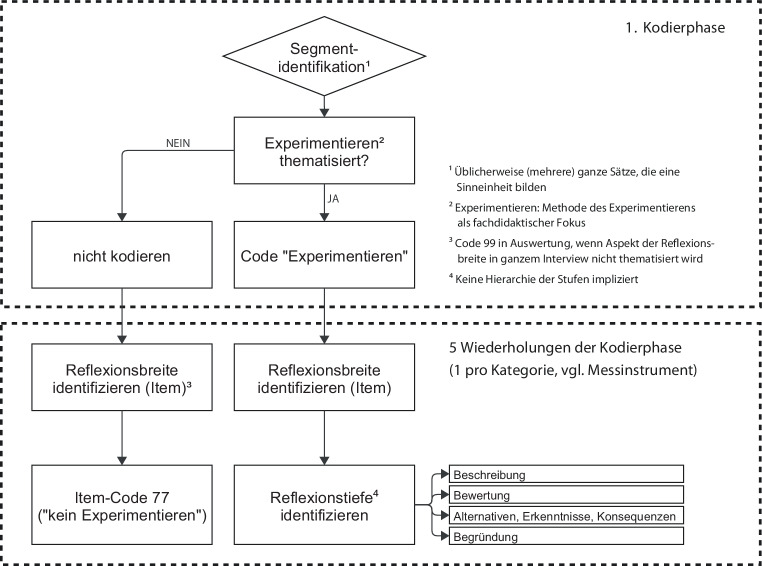
Ablauf des Kodierprozesses und die Vergabe von Codes für Reflexionsbreite und Reflexionstiefe

Die Transkriptionen wurden anhand des Messinstruments PURPUR‑R mit MAXQDA 2022 inhaltsanalytisch durch zwei Kodierende geratet (Mayring 2015). Ein entsprechendes Kodiermanual für die zwei Fachkontexte wurde deduktiv entwickelt und fortwährend induktiv ausgeschärft. Das Material wurde zwecks zuverlässiger Kodierung mit jeweils sechs Durchgängen pro Interview kodiert (Abb. 3). Dabei wurde in einem ersten Schritt kodiert, ob sich ein Interviewsegment auf das Lernziel des Experimentierens als Erkenntnismethode bezieht. In einem zweiten Schritt wurde kodiert, welche Items der Reflexionsbreite das Segment anspricht. In einem dritten Schritt wurde für die identifizierten Items die Reflexionstiefe bestimmt.

Wie bereits im theoretischen Rahmen erläutert, ist für eine vergleichbare Kodierung der Reflexion zweier Unterrichtseinheiten der Bezug auf ein gemeinsames Lernziel vor allem dann wesentlich, wenn wie in der vorliegenden Studie der Fokus auf fachdidaktische Aspekte gelegt wird, die mit Anforderungen an einen qualitativ hohen, sprich lernwirksamen, Unterricht verknüpft werden. Das Lernziel des Experimentierens als idealerweise hypothesengestützte, manipulative und kontrollierte Methode, um kausale Erkenntnisse über die Natur zu gewinnen, drückt sich im Rahmen der praktischen Umsetzung des Assel- und Tablettenexperiments in einer Vielzahl von Lerninhalten aus, die über reine Designfragen der Variablenkontrolle hinausgehen. Im Unterricht stellen sich unweigerlich eine Vielzahl von Fragen: Zum Beispiel, wie wird die abhängige Variable operationalisiert und gemessen? Wie wird dabei mit Messungenauigkeiten umgegangen? Wie wird die unabhängige Variable variiert und gemessen? Wie werden die Störvariablen kontrolliert oder, wenn das nicht möglich ist, ausbalanciert? Oder: Wie werden die Messdaten protokolliert, ausgewertet und hypothesenbezogen gedeutet? Die Erhebungen haben gezeigt, dass die Studierenden sich im Unterricht auf wenige Fragen zum facettenreichen Konstrukt des Experimentierens konzentrierten und andere Fragen ausblendeten. Dies wurde in den Kodierungen berücksichtigt.

Für das Training der zwei Kodierenden wurden 49 Interviews (40 %) auf Konsens kodiert. 73 Interviews (60 %) wurden nur von einem Kodierer bearbeitet. Davon wurden 12 Interviews (16 %) benutzt, um die Interrater-Reliabilitäten mittels Kappa (κ) nach Brennan und Prediger (1981) zu bestimmen. Diese erwiesen sich für jedes der 17 Items als akzeptabel (0,62–0,88), außer beim Item *Zielklarheit* (0,52) aufgrund der starken Asymmetrie der Codes (Onlinematerial A).

### Auswertung

Die Forschungsfragen 1 und 2 sind eng miteinander verknüpft. Beide erfordern eine Validierungsargumentation, die sowohl strukturelle als auch inhaltliche Überlegungen zum zu messenden Konstrukt einbezieht. Der Einfachheit halber werden die zwei Validierungsschwerpunkte aufgetrennt. Für die Beantwortung der Forschungsfrage 1 werden nur empirische Strukturanalysen der zwei Erhebungen genutzt (strukturelle Validierung). Für die Beantwortung der Forschungsfrage 2 werden die Ergebnisse zur Forschungsfrage 1 inhaltlich analysiert (inhaltliche Validierung).

Im Folgenden werden verschiedene Strukturanalysen des mit dem Messinstrument PURPUR‑R erhobenen Konstrukts der Reflexionsfähigkeit mit fachdidaktischem Fokus vorgestellt (Forschungsfrage 2). Dabei wird jede Analyse separat mit den beiden Messungen der Reflexionsfähigkeit, sprich der Messung mit Fachkontext „Asseln“ und der Messung mit Fachkontext „Tabletten“, durchgeführt. Anschließend werden die Ergebnisse verglichen (Forschungsfrage 1).

## Ergebnisse

### Prüfung der Eindimensionalität

Um die strukturelle Stabilität einschätzen zu können, müssen die Messungen der Reflexionsfähigkeiten über die beiden Fachkontexte hinweg verglichen werden. Hierzu ist eine gemeinsame Skala notwendig. Da das Messinstrument von Reflexionsfähigkeit als eindimensionalem Merkmal ausgeht, wird diese Annahme der Eindimensionalität zuerst mittels Reliabilitätsmaßen geprüft. Cronbachs α (Alpha) als das am häufigsten verwendete Reliabilitätsmaß geht von der Annahme aus, dass alle Items gleich stark mit der latenten Variable korrelieren, was nicht zwingend zutreffen muss (McNeish 2018; Revelle und Condon 2019; Sijtsma 2009; Wirtz 2023). Cronbachs α unterschätzt daher die Reliabilität in vielen Fällen (McNeish 2018). An Cronbachs α wird zudem die Abhängigkeit von der Itemanzahl kritisiert, denn trotz niedriger mittlerer Inter-Item-Korrelation können mit langen Tests hohe α‑Werte generiert werden (Hayes und Coutts 2020). Aufgrund der hohen Itemanzahl des Messinstruments PURPUR‑R und der Komplexität des zu messenden Konstrukts wird die Reliabilität zusätzlich mit McDonalds ω (Omega) abgesichert.^1^ Da Omega Total (ω_t_) konzeptionell Cronbachs α am ähnlichsten ist, wird dieses Maß berichtet. Die Reliabilitäten der Messung mit Fachkontext „Assel“ betragen α = 0,65; ω_t_ = 0,71; diejenigen für die Messungen mit Fachkontext „Tabletten“ α = 0,66; ω_t_ = 0,74. Als Cut-off-Wert von ω für eine akzeptable interne Konsistenz wird 0,7 vorgeschlagen (McNeish 2018). Die Skalenwerte deuten auf eine mögliche, aber diskussionswürdige strukturelle Stabilität und Eindimensionalität des Konstrukts hin. Die nicht vollends befriedigenden Resultate legen nahe, dass die Skalen für eine reliable eindimensionale Messung zu homogenisieren sind oder mehrdimensionale Strukturen des Konstrukts zu prüfen sind.

### Homogenisierung der Skalen

Bei der Homogenisierung einer Skala werden Items, welche die Fähigkeit numerisch ungenügend abbilden, inhaltlich begründet eliminiert. Da von einer strukturell stabilen, fachkontextübergreifenden Fähigkeit ausgegangen wird, werden bei beiden Messungen die gleichen Items entfernt. Eine Homogenisierung der Skalen hat auch Auswirkungen auf die Inhaltsvalidität. Eine Überanpassung der Skalen an die Daten bei gleichzeitig mangelhafter Inhaltsvalidität soll vermieden werden (Yousfi 2004). Wegen der inhaltlichen Komplexität des zu messenden Konstrukts wird von einer eher inhomogenen Skala ausgegangen, weshalb keine maximalen Reliabilitätswerte angestrebt werden.

Eine Homogenisierung der Skalen durch Entfernung der drei Items Schüler*innen-gerechte Konzepte (R23), *Emotionale Situierung* (R51) und *Zeit am Auftrag* (R54) ergibt für die Messung mit Fachkontext „Asseln“ eine Reliabilität von ω_t_ = 0,74 und für die Messung mit Fachkontext „Tabletten“ die Reliabilität ω_t_ = 0,78.

Die Eliminierung der drei Items lässt sich sowohl inhaltlich als auch aufgrund der Kodierung rechtfertigen. Das Item R23 erwies sich als schwierig objektiv zu kodieren. Das Item R54 stellte beim Kodieren entweder eine Präzisierung von R53 dar oder die kodierten Aussagen waren eher unspezifisch. Die Zuordnung war daher herausfordernd oder nicht möglich. R51 ist eher breit definiert und war in Kombination mit unspezifischen Aussagen ebenfalls schwierig objektiv zu kodieren.

### Konfirmatorische Überprüfung der Mehrdimensionalität

**Abb. 4:**
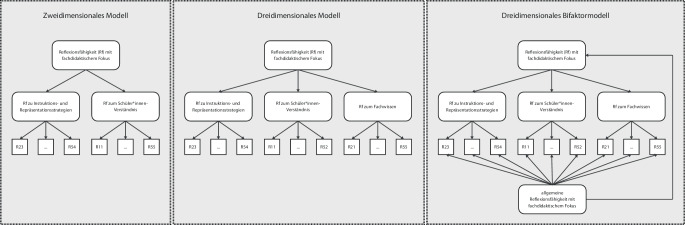
Vergleich der theoretischen Modelle mit unterschiedlichen Facetten. Die Einordnung der Items in die Facetten ist im Onlinematerial B zu finden

Zur Prüfung der Mehrdimensionalität wurden konfirmatorische Faktorenanalysen (CFA) mit theoriegestützten mehrdimensionalen Modellen gerechnet und verglichen (Abb. 4). Dazu wurden die 17 Items des Kodiersystems (s. Abb. 2) jeweils unterschiedlich in sogenannte Facetten gruppiert (s. Onlinematerial B). Den Facetten wurden zur Benennung die nachfolgend kursiv markierten Begriffe zugeordnet.

Der Vollständigkeit halber wurde die zur Reduktion der kognitiven Last beim Kodieren im Kodiersystem abgebildete Gliederung in fünf Facetten (s. Abb. 2) ebenfalls geprüft. Dieses Modell unterliegt dem Problem, dass ein Faktor mit lediglich zwei Items vorgegeben werden muss, und ergibt keine annähernd akzeptablen Werte. Es wird daher nicht weiterverfolgt.

Das zweidimensionale Modell unterscheidet die Facetten Reflexionsfähigkeit (Rf) zum Schüler*innen-*Verständnis *sowie *Reflexionsfähigkeit zu Instruktions- und Repräsentationsstrategien* und teilt die 17 Items einer der beiden Facetten zu. Diese Facetten umfassen die Anwendung von Wissen gemäß dem Vorschlag von Shulman (1986, 1987), welcher in fast allen PCK-Modellen Verwendung findet (Herzog 2019).

Für das dreidimensionale Modell wurde die Facette zum Schüler*innen-*Verständnis *aufgeteilt in eine zusätzliche Facette *Reflexionsfähigkeit zum Fachwissen* mit Items, die einen starken Fachwissensbezug haben, und eine reduzierte Facette zum Schüler*innen-Verständnis mit den restlichen Items. Je nach Modell wird das Fachwissen als eigenständig betrachtet oder dem PCK zugerechnet (Herzog 2019).

Als drittes Modell wurde ein dreidimensionales Bifaktormodell gerechnet, das neben den facettenspezifischen Reflexionsfähigkeiten die Wirkung einer *allgemeinen Reflexionsfähigkeit* annimmt.

Die CFA wurden getrennt nach Fachkontext für jedes Modell zweimal gerechnet.^2^ Tab. 1 zeigt die Fit-Werte.

**Tab. 1:** Vergleich der Modellgüte, getrennt nach Fachkontexten

		χ^2^	Df	*n*	*p*	CFI	RMSEA	SRMR
Fachkontext „Asseln“	Eindimensionales Modell	136,959	116	61	0,089	0,756	0,054	0,099
	Zweidimensionales Modell	136,690	115	61	0,082	0,747	0,056	0,098
	Dreidimensionales Modell	133,571	113	61	0,091	0,760	0,055	0,099
	Dreidimensionales Bifaktormodell	119,981	99	61	0,074	0,755	0,059	0,097
Fachkontext „Tabletten“	Eindimensionales Modell	139,138	116	61	0,071	0,828	0,057	0,103
	Zweidimensionales Modell	137,220	115	61	0,077	0,835	0,056	0,105
	Dreidimensionales Modell	132,223	113	61	0,104	0,858	0,053	0,107
	Dreidimensionales Bifaktormodell	118,359	99	61	0,090	0,857	0,057	0,092

Nach Hu und Bentler (1999) ist der RMSEA bei allen Modellen ausreichend (< 0,06), der CFI (≥ 0,95) und der SRMR (< 0,08) jedoch ungenügend. Nach Moosbrugger und Kelava (2020) sind sämtliche SRMR-Werte an der Grenze zum akzeptablen Bereich (≤ 0,10), jedoch weit weg davon, als gut gewertet zu werden (≤ 0,05). Die Unterschiede in den Werten der Modellgüte zwischen den Fachkontexten erweisen sich als nicht signifikant, womit alle Modelle aufgrund der gegebenen strukturellen Stabilität eine ähnlich hohe theoretische Plausibilität aufweisen. Aufgrund der ungenügenden Werte der Modellgüte selbst überzeugt empirisch keines der drei gerechneten Modelle.

### Explorative Prüfung der Mehrdimensionalität

**Abb. 5:**
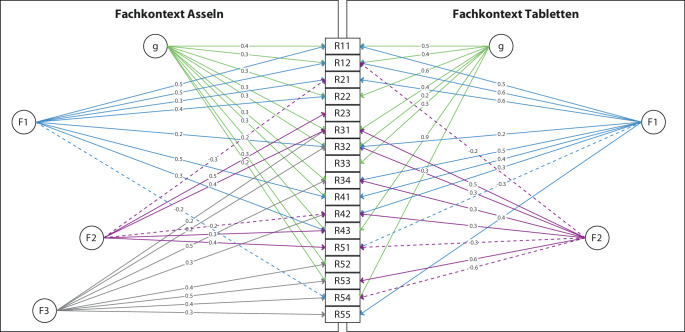
EFA (Verfahren: hierarchische Faktorenanalyse mit obliquer Rotation) des Instruments, getrennt nach Fachkontext

Als Alternative zu konfirmatorischen Strukturanalysen wird abschließend eine exploratorische Analyse eines Bifaktormodells vorgestellt. Die Ergebnisse der für jeden Fachkontext separat durchgeführten explorativen Faktorenanalysen (EFA) werden in der Abb. 5 gegenübergestellt. Die Kästchen mit R11 etc. repräsentieren die 17 manifesten Items. Kreise mit F1, F2, F3 sind die extrahierten latenten Faktoren. g bezeichnet einen allgemeinen Faktor. Gestrichelte Linien deklarieren negative Faktorladungen.

Die faktorielle Struktur ist zwischen den zwei Messungen in großen Teilen vergleichbar. Dennoch sind Differenzen ersichtlich, die es zu diskutieren gilt. Einerseits werden nicht gleich viele Faktoren extrahiert. Andererseits korrelieren einige Items mit relevanten Ladungen negativ mit Faktoren. Die Strukturen der EFA lassen sich (aufgrund der CFA erwartungsgemäß) nicht eindeutig in die Facetten einteilen. Es zeigt sich inhaltlich zudem keine interpretierbare Struktur. Die explorative Analyse ergibt somit keine akzeptable stabile mehrdimensionale Struktur des Konstrukts der Reflexionsfähigkeit.

## Diskussion

Mit den beschriebenen Analysen werden die gestellten Forschungsfragen beantwortet, wobei zuerst die Frage der strukturellen Stabilität geklärt wird. Darauf aufbauend wird die Dimensionalität betrachtet.

### F1: Inwiefern kann die Reflexionsfähigkeit in Bezug auf die Vermittlung eines kontrollierten Lernziels über zwei Unterrichtseinheiten mit derselben Klasse strukturell stabil erfasst werden?

Die beiden Messungen der Reflexionsfähigkeit erweisen sich prima facie als strukturell stabil, wenn von einem eindimensionalen Konstrukt ausgegangen wird. Die Skalierungen ergeben vergleichbare und akzeptable Reliabilitäten, die im Bereich oder oberhalb dessen liegen, was bei Messungen ähnlicher Konstrukte bisher ebenfalls erreicht wurde (vgl. z. B. Wöhlke und Höttecke 2022). Als möglicherweise strukturell stabil zeigten sich auch mehrdimensionale bzw. multifaktorielle Modelle, die jedoch an ungenügenden Werten der Modellgüte scheitern. Diese Aussage relativiert sich hingegen insofern, als dass die Stichprobengröße für aussagekräftige Faktorenanalysen gering ist (Klopp 2010). Es sei betont, dass für die Güte der Messung neben der möglichen Skalierbarkeit auch die Tatsache spricht, dass die Daten aus einer ausreichend objektivierten Kodierung hervorgingen.

Die konstatierte strukturelle Stabilität der eindimensionalen Skalierung kann aufgrund des Erhebungsdesigns mit den zwei methodisch vergleichbaren Experimenten aus unterschiedlichen Disziplinen als Fachkontextunabhängigkeit der Fähigkeitsmessung interpretiert werden, nicht jedoch als Lernzielunabhängigkeit. Diese Feststellung ist für die Plausibilisierung und Wertung des Ergebnisses aus zweierlei Gründen relevant. Zum einen sind viele mit dem Messinstrument PURPUR‑R kodierte Items in erster Linie vom Lernziel und nur in zweiter Linie vom Fachkontext abhängig. Einige Items sind sogar unabhängig vom Fachkontext. Zum Beispiel geht es beim Item *kognitiver Anspruch* (R52) um Instruktionen jeglicher Art, die das Potenzial haben, Schüler*innen zu aktivieren, über das Lernziel des Experimentierens nachzudenken (s. Onlinematerial D). Der Fachkontext spielt für die Konzipierung solcher Instruktionen nur eine untergeordnete Rolle. Da das Lernziel bei den zwei Messungen kontrolliert wurde, fällt der inhaltlich gewichtigere Einflussfaktor, der strukturell einen Unterschied zwischen den Messungen machen könnte, weg. Zum anderen wird dieser Effekt durch die Kodierung verstärkt (s. Abb. 3), da Interviewsegmente, die keine erkennbare Relevanz für die Erreichung des Lernziels des Experimentierens haben, für die Bewertung der Reflexionsfähigkeit ausgeschlossen wurden.

### F2: Sofern die Reflexionsfähigkeit strukturell stabil erfasst werden kann, welche inhaltlich valide Dimensionalität weist die Reflexionsfähigkeit auf?

Im Rahmen einer Validierung ist mindestens ein zweiter Blick auf die Ergebnisse notwendig. Die Tatsache, dass beide Messungen auf die gleiche Weise eindimensional skaliert werden können, ist zwar ein Beleg für die Möglichkeit, dass damit auch eine latente Fähigkeit gemessen wird. Es stellen sich dennoch die Fragen, inwiefern vor dem Hintergrund der Modellierung mit 17 vielfältigen, vorwiegend fachdidaktischen Aspekten der Reflexion und dem gewählten Erhebungsdesign (a) die Eindimensionalität zusammen mit der Art der Homogenisierung und (b) das Fehlen einer alternativen mehrdimensionalen Struktur eine sinnvolle Interpretation der Fähigkeit zulassen (vgl. Schreiber und Gut 2022). Im Folgenden soll zuerst auf die Frage (b), dann auf die Frage (a) eingegangen werden.

Inwieweit spricht das Fehlen einer multifaktoriellen Struktur für die Interpretation, dass mit dem Instrument PURPUR‑R eine latente, interpretierbare Fähigkeit gemessen wird? Die Beantwortung dieser Frage muss von den Erwartungen ausgehen, die man aufgrund der Modellierung und Theorie an das Messinstrument stellt. Das hier zu validierende Instrument wurde analog zu einem bereits validierten Instrument für Planungsfähigkeit entwickelt, das auf der Denkstruktur der didaktischen Rekonstruktion aufbaut und zwischen Aspekten, die die Analyse der Ausgangslage vor dem Unterricht betreffen, und Aspekten, die die Konstruktion bzw. Inszenierung des geplanten Unterrichts betreffen, eine zweidimensionale Struktur aufweist (Tardent 2020). Eine solche Struktur konnte für die Reflexionsfähigkeit nicht nachgewiesen werden. Es ließe sich jedoch auch dagegenhalten, dass Aspekte, die bei der Planung die Analyse oder die Konstruktion betreffen, bei der Reflexion des umgesetzten Unterrichts ihren distinguierten Charakter verlieren, auch wenn die Planung in einer Unterrichtsreflexion thematisiert werden kann.

Qualitätsvolles Reflektieren fördert den Erwerb von relevantem Professionswissen. Zudem besteht die Theorie, dass relevantes Professionswissen ursächlich für qualitätsvolles Reflektieren ist. Diese beiden Umstände begründen die Hypothese, dass die Vielfalt der mit dem Messinstrument PURPUR‑R erhobenen Aspekte beim Reflektieren, die unterschiedlich auf fachliche und fachdidaktische Wissensbestände referieren, die Struktur dieser Wissensbestände zu einem gewissen Grad abbilden. Auch gegen diese Annahme ließe sich mit dem Hinweis argumentieren, dass die einzelnen Aspekte nicht ausschließlich einen auf der Achse von Fachwissen, fachdidaktischem Wissen und allgemeindidaktischem Wissen distinguierten Wissensbestand ansprechen. Die gewählte Modellierung „verwäscht“ sozusagen eine mögliche allgemein bestehende Wissensstruktur. Die gemessene Reflexionsfähigkeit lässt sich so als übergeordnete allgemeine Fähigkeit interpretieren, naturwissenschaftlichen Unterricht zu reflektieren.

Dieses Ergebnis erscheint auch vor dem Hintergrund der Differenzierungshypothese von Fähigkeitsstrukturen (Köller und Parchmann 2012) nicht unplausibel, wonach komplexe Konstrukte wie die Reflexionsfähigkeit bei Studierenden, wenn überhaupt als Konstrukt ausgebildet, weniger ausdifferenziert vorliegen als bei Expert*innen. Erst durch die Professionalisierung bilden sich komplexe Strukturen eines Konstrukts aus. Eine Hypothese, die es für die Reflexionsfähigkeit empirisch zu überprüfen gilt.

Inwiefern lässt die eindimensionale, homogenisierte Skalierung die Interpretation zu, dass mit dem Instrument PURPUR‑R eine latente, interpretierbare Fähigkeit gemessen wird? Wie bereits erwähnt, ist die Eindimensionalität der sich als strukturell stabil erwiesenen Skalierung per se nicht problematisch. Die Homogenisierung erfordert hingegen eine weitergehende Diskussion.

Die Tatsache, dass die Skalierungen in beiden Fachkontexten durch die gleiche Homogenisierung konsistenter werden, könnte dahin gehend gedeutet werden, dass es in dem vorerst als übergeordnete, allgemeine Reflexionsfähigkeit interpretierten eindimensionalen Konstrukt, zumindest empirisch eine ausgeprägte Teilstruktur gibt. Es stellt sich dabei die Frage, wieso gewisse Aspekte (Items), konkret die Items Schüler*innen-gerechte Konzepte (R23), Emotionale Situierung (R51) und *Zeit am Auftrag (R54)*, beim Homogenisieren aus dieser Teilstruktur fallen. Aus inhaltlicher Sicht drängt sich keine Erklärung dafür auf, warum gerade diese drei heterogen erscheinenden Items nicht zum restlichen Gesamtkonstrukt beitragen sollen. Ein gangbarer Erklärungsansatz könnte demgegenüber das Messinstrument selbst liefern.

Zwar wurden bei den beiden Messungen durch die Kontrolle der Klasse, Lernziele und entsprechender Kodierung (vgl. Diskussion zur Forschungsfrage F1) sowie durch die Vorgabe von Experimentiermaterial für den Unterricht, der später reflektiert werden sollte, die Anforderungssituationen der beiden Messungen der Reflexionsfähigkeit, bestmöglich vergleichbar gemacht. Dennoch führen Unwägbarkeiten, die realem Unterricht im Schulfeld eigen sind, zu viel Variabilität bei den Stimuli der beiden Messungen. Darüber hinaus wurden beim Messinstrument PURPUR‑R individuelle Reflexionsinterviews bewertet, wobei im Interview die fachdidaktischen Items nicht namentlich angesprochen, jedoch teilweise implizit mit Fragen getriggert wurden (s. Interviewleitfaden im Onlinematerial C). Die Inhalte der Reflexion wurden damit durch die selektive Wahrnehmung (*Noticing)* der Studierenden gefiltert. Die Kodierung erwies sich deshalb auch nicht als einfach, weil viele Konzepte, die mit den verschiedenen Items verknüpft werden, in den Interviews nur sehr selten namentlich ausgesprochen wurden. Es kann daher nicht ausgeschlossen werden, dass es sich bei der Homogenisierung, die sich bei beiden Messungen als die konsistentere Variante erwies, um ein Artefakt des Messinstruments handelt.

Im Sinne einer Bewertung des Messinstruments PURPUR‑R durch die Autor:innen wird abschließend der Standpunkt vertreten, dass die bisherige Validierung der Messungen Anlass gibt zur begründeten Hypothese, dass mit der Gesamtheit aller 17 Items eine eindimensionale übergeordnete, fachdidaktisch ausgerichtete Reflexionsfähigkeit erfasst werden kann. Die Messungen erweisen sich zumindest über zwei Wiederholungen zu unterschiedlichen Fachkontexten aus der Biologie und der Chemie mit für die spezifische Art des Messinstruments (Reflexionsinterview zu selbst erteiltem Unterricht) akzeptabler Konsistenz (ω_t_ > 0,70) als strukturell stabil. Die Hypothese macht jedoch auch weitere Validierungen notwendig, die in den folgenden zwei Abschnitten angesprochen werden.

## Limitationen

Die Beantwortung der Forschungsfragen erfährt in Bezug auf ihre Aussagekraft und Verallgemeinerbarkeit Limitationen, die durch die Modellierung der Reflexionsfähigkeit im Messinstrument PURPUR‑R, das Erhebungsdesign, das Forschungsdesign und die Stichprobe bedingt werden.

Mit dem hier vorgestellten Messinstrument werden in Bezug auf die Konzeption einer Reflexionsfähigkeit a priori-Setzungen vorgenommen, die sich in den 17 kodierten fachdidaktischen Items widerspiegeln. Diese decken ein breites Feld an Unterrichtsaspekten ab, wobei einige in Bezug auf die Reflexionsfähigkeit anspruchsvolle Konzepte behandeln (vgl. analoge Diskussion zur Modellierung experimenteller Kompetenzen in Gut und Mayer 2018). Dazu gehört u. a. die Entscheidung, allgemeindidaktisch-pädagogische Aspekte der Unterrichtsreflexion nicht zu erheben, die für faktorielle Analysen mehr Kontrast geboten hätten. Im Rahmen dieser Setzungen scheint das Konzept der Reflexionsfähigkeit ein psychometrisch messbares, eindimensionales Konstrukt zu sein, wobei die Stichprobengröße nicht ausreicht, um komplexere Strukturen des Konstrukts aufzudecken. In diesem Sinne besteht die Beantwortung von Forschungsfrage 2 in der bloßen Klärung der strukturellen Validität eines spezifischen Messinstruments.

Die Aussagekraft der Ergebnisse sollte zudem vor dem Hintergrund der Frage relativiert werden, in welcher Schärfe die Reflexionsfähigkeit der Studierenden durch den Messprozess erfasst wurde bzw. inwieweit der Reflexionsprozess vom gewählten Forschungs- und Erhebungsdesign beeinflusst wurde (Wyss 2013). Die Messungen der Reflexionsfähigkeit basieren im Projekt PURPUR auf vorwiegend monologischen und geschlossenen Unterrichtsreflexionen zu einem selbst erteilten Unterricht, die mit semistrukturierten Leitfadeninterviews generiert wurden. Die Interviewfragen triggern implizit das Nachdenken über bestimmte, für die Kodierung relevante fachdidaktische Aspekte des Unterrichts. Dadurch wird in der Messung automatisch ein Gewicht auf das Erkennen von diskussionswürdigen Situationen gelegt (s. *Noticing*). Eine Generalisierung der Ergebnisse darf aufgrund der Situationsabhängigkeit des Messprozesses (Unkontrollierbarkeit der diskutablen Unterrichtsereignisse, implizites Triggern des Nachdenkens über fachdidaktische Aspekte sowie die Fachkontext- und Lernzielabhängigkeit der Kodierung) nur mit Vorsicht geschehen.

Diese Generalisierung betrifft auch die Übertragung der Ergebnisse auf andere Stichproben. Durch die Wahl einer anfallenden Stichprobe muss von einer tendenziell höheren Motivation für die Thematik ausgegangen werden. Ein Einfluss auf die Reflexionsbereitschaft als persönlicher Prozess ist nicht auszuschließen (Wyss 2013). Zur Kontrolle wurden für ein Hintergrundmodell entsprechende Daten mittels Fragebogen erhoben. Ein erkennbarer Einfluss ist nicht ersichtlich, wird aber in künftigen Publikationen genauer untersucht.

Zu den durch das Forschungsdesign bedingten Limitationen gehört die eingeschränkte Verallgemeinerbarkeit der Ergebnisse zur strukturellen Stabilität. Es ist unklar, inwieweit die strukturelle Stabilität bei der Messung der Reflexionsfähigkeit Bestand hält, wenn Unterricht zu unterschiedlichen Lernzielen verglichen wird. Variierende Lernziele erfordern beim Messinstrument PURPUR‑R auf der Ebene des Kodiermanuals tiefgreifendere situative Anpassungen als die Variation der Fachkontexte.

## Ausblick und Desiderata

Die Studie zeigt, dass eine detaillierte Erfassung einer Reflexionsfähigkeit mit fachdidaktischem Fokus anhand der zwei Progressionsdimensionen Reflexionsbreite und Reflexionstiefe mit dem Messinstrument PURPUR‑R in praxisnahen Unterrichtssituationen – sprich ökologisch valide – und mit Studierenden möglich ist. Die Reflexionsfähigkeit kann trotz einer sehr geringen Stichprobengröße und des als unscharf anzunehmenden Messprozesses als psychometrisches, zumindest eindimensionales Konstrukt vermutet werden. Die Herausforderung, die in der Literatur angenommenen Dimensionen in den Daten zu finden, unterstützt die Annahme, dass es sich bei der Reflexionsfähigkeit um ein vielseitiges Konstrukt handelt und Reflexionsanlässe stark von der jeweiligen Situation geprägt sein könnten. Dieser Beitrag stellt daher die Frage in den Raum, inwiefern von einer Reflexionsfähigkeit gesprochen werden kann, wenn keine strukturell stabilen Skalen geschaffen werden können. Zwar wurde in der vorliegenden Untersuchung eine stabile Skala gefunden, allerdings überzeugt deren Inhaltsvalidität nicht vollends. Inwieweit sich durch stärker fachdidaktisch fokussierte Erhebungsmethoden, bei größeren Stichproben und im Vergleich zu erfahreneren Lehrkräften komplexere, plausible Strukturen des Konstrukts zeigen, müsste durch weitergehende Studien untersucht werden (Desiderat 1).

Die Studie legt nahe, dass die Reflexionsfähigkeit mit dem Messinstrument PURPUR‑R über zwei bezüglich des Lernziels vergleichbare Unterrichtssituationen hinweg strukturell stabil erfassbar scheint. Inwieweit die strukturelle Stabilität über mehr als zwei Messungen und Unterricht zu unterschiedlichen Lernzielen Bestand hält, lässt sich anhand der Studie PURPUR‑R nicht ableiten. Dieser Nachweis, der die Durchführung einer weitergehenden Generalisierbarkeitsstudie bedingt (Desiderat 2), wäre notwendig, um von einer Reflexionsfähigkeit sprechen zu können.

Im Hinblick auf die Professionalisierung von Studierenden stellt sich die Frage, inwieweit die Reflexionsfähigkeit durch das Professionswissen und weitergehende Personenmerkmale erklärt wird (Desiderat 3). Eine erste Antwort auf diese Frage kann beispielsweise im Rahmen des Projekts PURPUR gegeben werden.

## Supplementary Information

**Onlinematerial A** Übersicht der Inter-Kodierer-Reliabilitäten für das Messinstrument PURPUR‑R

**Onlinematerial B** Zuordnung der Items zu den Modellen inklusive Übersicht zu den Kurzcodes der Items

**Onlinematerial C** Interview-Leitfaden für die Reflexionsgespräche

**Onlinematerial D** Übersicht der Item-Definitionen aus dem Kodiermanual

## Footnotes

1 Berechnung mit omega-Funktion, R‑Package psych, Version 2.3.9.

2 RStudio, Version 2023.06.1 Build 524; R‑Package Lavaan, Version 0.6-16.
