# Supplementary material for: Assessment of Dimensionality and Structural Stability of Pre-service Science Teachers’ Ability to Engage in Reflections
Source: Z Didakt Nat Wiss. 2025 Nov 27;31(1):12. [Article in German] doi: 10.1007/s40573-025-00186-7 (PMC12660408; doi:10.1007/s40573-025-00186-7)
Supplement: Supplementary file 1 — Onlinematerial A Übersicht der Inter-Kodierer-Reliabilitäten für das Messinstrument PURPUR‑R [file 40573_2025_186_MOESM1_ESM.pdf]

Onlinematerial A: Reliabilitätsanalysen

Inter-Kodierer-Reliabilität (ICR) Kappa (Berechnung nach Brennan und Prediger 1981)

Reflexionsbreite: Übereinstimmung der Codes inkl. 77 und 99, pro Item

Schüler:innenvorstellungen*	.77
Aktivierung und / oder Erhebung	.79
Umgang	.62
Fachliche Klärung*	.81
Konzeptionelle Korrektheit	.83
Sachgerechte Sprache	.70
Schüler:innen-gerechte Konzepte	.75
Unterrichtsziele*	.83
Zielauswahl	.76
Zielorientierung	.79
Zielklarheit	.52
Zielerreichung	.88
Reflexivität*	.81
Prozessreflexion	.78
Ergebnisreflexion	.75
Begriffsbildung	.81
Didaktische Strukturierung*	.89
Emotionale Situierung	.86
Kognitiver Anspruch	.86
Seq. und Strukt. des Lernprozesses	.87
Zeit am Auftrag	.86
Material	.87

** ICR für aggregierte Subcodes*

Reflexionstiefen: Übereinstimmung der Codes über alle Items

77*	.78
Experimentelles Handeln (ExpH)	.93
Beschreibung*	.85
Bewertung*	.86
Alternativen, Erkenntnisse, Konsequenzen*	.85
Begründung*	.86

** ICR für gleichwertige Indikatoren*
