# Supplementary material for: Assessment of Dimensionality and Structural Stability of Pre-service Science Teachers’ Ability to Engage in Reflections
Source: Z Didakt Nat Wiss. 2025 Nov 27;31(1):12. [Article in German] doi: 10.1007/s40573-025-00186-7 (PMC12660408; doi:10.1007/s40573-025-00186-7)
Supplement: Supplementary file 2 — Onlinematerial B Zuordnung der Items zu den Modellen inklusive Übersicht zu den Kurzcodes der Items [file 40573_2025_186_MOESM2_ESM.pdf]

Onlinematerial B: Zuordnung der Items zu den Modellen

Kürzel	Kategorie	Item
R11	SuS-Vorstellungen	Aktivierung und/oder Erhebung
R12	SuS-Vorstellungen	Umgang
R21	Fachliche Klärung	Konzeptionelle Korrektheit
R22	Fachliche Klärung	Sachgerechte Sprache
R23	Fachliche Klärung	Schüler:innen-gerechte Konzepte
R31	Unterrichtsziele	Zielorientierung
R32	Unterrichtsziele	Zielklarheit
R33	Unterrichtsziele	Zielauswahl
R34	Unterrichtsziele	Zielerreichung
R41	Reflexivität	Prozessreflexion
R42	Reflexivität	Ergebnisreflexion
R43	Reflexivität	Begriffsbildung
R51	Didaktische Strukturierung	Emotionale Situierung
R52	Didaktische Strukturierung	Kognitiver Anspruch
R53	Didaktische Strukturierung	Sequenzierung und Strukturierung
R54	Didaktische Strukturierung	Zeit am Auftrag
R55	Didaktische Strukturierung	Material

Begriffserklärungen:

KISR Facette zu fachdidaktischer Reflexionsfähigkeit zu Instruktions- und Repräsentationsstrategien. Basierend auf *Knowledge of instructional strategies and representations (KISR)* von Shulman (1986).

KSU Facette zu fachdidaktischer Reflexionsfähigkeit zum Schüler*innen-Verständnis. Basierend auf *Knowledge of students' understanding (KSU)* von Shulman (1986).

CK Facette zu fachdidaktischer Reflexionsfähigkeit zum Fachwissen. Basierend auf *Content knowledge (CK)* von Shulman (1986).

Eindimensionales Modell: 1 Faktor = ExpH

EXPH =~ R11 + R12 + R21 + R22 + R23 + R31 + R32 + R33 + R34 + R41 + R42 + R43 + R51 + R52 + R53 + R54 + R55

R53 ~~ R54 # *Abhängigkeit durch Kodierregel – meist eine Präzisierung*

R22 ~~ R43 # *Starke gemeinsame Abhängigkeit von Sprache*

R11 ~~ R12 # *Starke gemeinsame Abhängigkeit von Schüler*innen-Vorstellungen*

Zweidimensionales Modell: 2 Faktoren = KISR, KSU (CK in KSU enthalten)

KISR =~ R23 + R31 + R53 + R54

KSU =~ R11 + R12 + R32 + R33 + R34 + R41 + R42 + R51 + R52 + R21 + R22 + R43 + R55

R53 ~~ R54

R22 ~~ R43

R11 ~~ R12

Dreidimensionales Modell: 3 Faktoren = KSU, KISR, CK

KISR =~ R23 + R31 + R53 + R54

KSU =~ R11 + R12 + R32 + R33 + R34 + R41 + R42 + R51 + R52

CK =~ R21 + R22 + R43 + R55

R53 ~~ R54

R22 ~~ R43

R11 ~~ R12

Dreidimensionales Bifaktormodell: 3+1 Faktoren = KSU, KISR, CK + genereller Faktor

KISR =~ R23 + R31 + R53 + R54

KSU =~ R11 + R12 + R32 + R33 + R34 + R41 + R42 + R51 + R52

CK =~ R21 + R22 + R43 + R55

R53 ~~ R54

R22 ~~ R43

R11 ~~ R12

g =~ R11 + R12 + R21 + R22 + R23 + R31 + R32 + R33 + R34 + R41 + R42 + R43 + R51 + R52 + R53 + R54 + R55
