# Supplementary material for: Assessment of Dimensionality and Structural Stability of Pre-service Science Teachers’ Ability to Engage in Reflections
Source: Z Didakt Nat Wiss. 2025 Nov 27;31(1):12. [Article in German] doi: 10.1007/s40573-025-00186-7 (PMC12660408; doi:10.1007/s40573-025-00186-7)
Supplement: Supplementary file 3 — Onlinematerial C Interview-Leitfaden für die Reflexionsgespräche [file 40573_2025_186_MOESM3_ESM.pdf]

Onlinematerial C: Interview-Leitfaden

1. Ist Ihnen in Ihrem Unterricht etwas besonders aufgefallen? Wenn ja, was und warum?
2. Womit sind Sie bei Ihrem Unterricht besonders zufrieden und wo eher unzufrieden?
3. Haben Sie nach dem Planungsgespräch an Ihrem Unterricht, so wie er dort besprochen wurde, noch etwas geändert? Wenn ja, was und warum?
4. Haben Sie Ihren Unterricht gegenüber der Planung spontan verändert? Wenn ja, was und warum?
5. Beurteilen Sie den, durch Ihre Lektion angelegten und gestalteten Lernprozess in Bezug auf die Erreichung des primären Lernziels gemäß Auftrag. Was würden Sie an der Gestaltung des Lernprozesses beibehalten und was würden Sie ändern?
6. Beurteilen Sie die Erreichung der angestrebten Lernziele im Unterricht. Wie schätzen Sie den Lernzuwachs in Bezug auf die Lernziele ein?
7. Wo haben Sie während dem Unterrichten bei einzelnen Lernenden lückenhafte Konzepte bzw. lückenhaftes Experimentieren entdeckt und wie sind Sie damit umgegangen?
8. Inwiefern haben die Schülerinnen und Schüler über ihr Experimentieren nachgedacht?
9. Beurteilen Sie die Angemessenheit Ihrer Aufgabenwahl und deren Umsetzung. Wo sind Ihnen Über- oder Unterforderungen bei beispielsweise einzelnen Lernenden aufgefallen?
10. Inwiefern sind Sie mit der Differenzierung nach den Fähigkeiten und Fertigkeiten der Schülerinnen und Schüler in Ihrem Unterricht zufrieden oder unzufrieden?
11. Bei welchen Themen und Situationen waren Sie selbst fachlich unsicher oder sind es noch? Wie sind Sie damit umgegangen?
12. Wenn Sie freie Wahl gehabt hätten, würden Sie anderes Material/andere Geräte wählen? Wenn ja, warum?
13. Wenn Sie die Doppellektion noch einmal mit derselben Planung durchführen würden, was würden Sie im Unterricht anders machen? Und warum?
14. Sie müssen weiterhin das experimentelle Handeln als Lernziel verfolgen in dieser Frage: Was würden Sie an Ihrer Planung grundsätzlich verändern, wenn Sie diese Doppellektion noch einmal durchführen könnten?
15. *Nur bei 1. Interview:* Gibt es Erkenntnisse aus Ihrem Unterricht, die Sie nun für die Planung und Durchführung der zweiten Lektion/weiterer Lektionen berücksichtigen werden?
16. *Nur bei 2. Interview:* Gibt es Erkenntnisse aus Ihrem Unterricht, die Sie nun für die Planung und Durchführung von weiteren Lektionen berücksichtigen werden?
17. *Nur bei 2. Interview*: Gibt es für Sie Unterschiede zwischen der ersten videografierten Unterrichtseinheit und der zweiten? Wenn ja, welche und weshalb?
18. Gibt es sonst noch etwas, das Sie als Nachtrag auf eine der gestellten Fragen oder ganz allgemein anmerken möchten?

Aufrechterhaltungsfragen

- Können Sie dazu eine Situation aus dem Unterricht nennen?
- Können Sie dazu ein Beispiel einer Schülerin/eines Schülers oder einer Schülergruppe nennen?
- Wo finden Sie das in der schriftlichen Planung?
- Können Sie das beschreiben?
- Können Sie mir das erläutern?
- Können Sie das noch weiter ausführen?
- Können Sie das präzisieren?
- Kommen wir nochmals zur Frage zurück…
- Was meinen Sie genau mit dem Begriff xy?
- Können Sie das Gesagte zusammenfassen? (Sofern nicht klar wurde, was gemeint war.)
- Können Sie zu dem Begriff xy etwas sagen? (Aufgreifen von nicht beantworteten Fragen)
