# Supplementary material for: Assessment of Dimensionality and Structural Stability of Pre-service Science Teachers’ Ability to Engage in Reflections
Source: Z Didakt Nat Wiss. 2025 Nov 27;31(1):12. [Article in German] doi: 10.1007/s40573-025-00186-7 (PMC12660408; doi:10.1007/s40573-025-00186-7)
Supplement: Supplementary file 4 — Onlinematerial D Übersicht der Item-Definitionen aus dem Kodiermanual [file 40573_2025_186_MOESM4_ESM.pdf]

Onlinematerial D: Item-Definitionen aus Kodiermanual

Hinweis: Bei diesem Exzerpt handelt es sich lediglich um die Item-Definitionen. Das vollständige Kodiermanual umfasst detaillierte Ausschärfungen für die Reflexionstiefen sowie Ankerbeispiele und ist auf Anfrage über die Korrespondenzadresse bei den Autoren erhältlich. Es werden folgende Abkürzungen verwendet: LK = Lehrkraft, SuS = Schüler*innen, ExpH = experimentelles Handeln als Lernziel

Schüler:innenvorstellungen<br>**Aktivierung und / oder Erhebung**
**Die LK macht Aussagen dazu, inwiefern SuS-Vorstellungen zum ExpH aktiviert, erhoben oder festgestellt wurden.**<br>- **Aktivieren:** LK macht Aussagen dazu, inwiefern sie Handlungen vorgenommen hat oder festgestellt hat, damit/dass die SuS über ihre Vorstellungen/ihr Vorwissen nachdenken.<br>- **Erhebung:** LK macht Aussagen dazu, inwiefern sie probiert hat, Situationen zu schaffen, damit die SuS ihre Vorstellungen zeigen/äußern und die LK/die anderen diese wahrnehmen können.<br>- **Feststellung:** LK macht Aussagen dazu, inwiefern sie SuS-Vorstellungen (lückenhaft oder nicht) festgestellt hat.<br><br>○ SuS-Vorstellungen zum ExpH sind bspw. Wissen/Vorstellung über den Experimentierzyklus/Erkenntnisgewinnungsprozess; Notwendigkeit für geplantes Vorgehen; Notwendigkeit von Messwiederholungen etc.<br>○ SuS-Vorstellungen können auch Bezüge zu vorhandenem/fehlendem Vorwissen als Basis für Vorstellungen sein (je nach Art des Vorwissens ExpH oder 77) (Beispiel: „Die SuS haben Messwiederholung/Asseln nicht gekannt“) (Definition SuS-Vorstellungen: Adamina et al. 2018, 7-8[1]).<br>○ SuS-Vorstellungen können Bezüge zu vorhandenem/fehlendem Verständnis von, für Vorstellungen relevante, Begriffen sein.<br><br>**Hinweis:** Aktivierung/Erhebung/Feststellung kann in jeder Phase des Unterrichts, geplant/spontan oder im Plenum/in Gruppen/einzeln stattfinden (Beispiel: Rückfrage bei einzelnen Gruppen zu Experimentierüberlegungen in Arbeitsphase).<br><br>**Hinweis:** Es ist gut darauf zu achten, wo SuS-Vorstellungen tatsächlich zum ExpH sind und wo es eher in den Fachkontext A/T übergeht.

Schüler:innenvorstellungen<br>**Umgang**
**Die LK macht Aussagen zum Umgang mit den festgestellten oder erwarteten SuS-Vorstellungen.**<br>Nebst dem Umgang mit den spontan festgestellten SuS-Vorstellungen im Unterricht (z. B. auch fehlendes Vorwissen) ist hier auch der Umgang mit zu erwartenden oder ausgebliebenen SuS-Vorstellungen (z. B. aus Planung) relevant.<br><br>Möglichkeiten zum Umgang können z. B. Paraphrasierung von SuS-Vorstellungen, Aufnahme der SuS-Vorstellungen im Unterricht und Ausrichtung des Unterrichts danach, bewusstes Ignorieren/Vertagen etc. sein.<br><br>**Hinweis:** In seltenen Fällen wird trotz fehlender „Aktivierung und / oder Erhebung“ aus dem Kontext klar, dass sich der Umgang auf eine SuS-Vorstellung bezieht. In diesen Fällen wird „Umgang“ kodiert.

Fachliche Klärung<br>**Konzeptionelle Korrektheit**
**Die Bezüge zur konzeptionellen Korrektheit von ExpH können sich auf die LK selbst oder die SuS beziehen. Hierbei werden die Korrektheit, Inkorrektheit oder Unsicherheit/Lücken in Bezug auf Konzepte und Strategien zum ExpH angesprochen.**<br><br>Die Sprache (z. B. falsche Verwendung von Fachbegriffen) ist hierbei ausgenommen. Es geht nur um (nicht sprachliche) Konzepte.<br><br>**Hinweis:** Der Bezug zur Korrektheit/Inkorrektheit muss für ExpH klar und wenn möglich explizit sein. Ausschließliche Beschreibungen der LK, wie die Erreichung der konzeptionellen Korrektheit sichergestellt wird, werden nicht kodiert.<br><br>**Hinweis:** Spricht die LK lediglich an, dass es keine fachlichen Unsicherheiten/Lücken gegeben hat, dann wird dies mit 77 kodiert.<br><br>**Hinweis:** Es kann auch die korrekte Einhaltung von Regeln wie bspw. Laborsicherheit als Konzept angesprochen werden (z. B. „[…] wenn man experimentiert, gibt es die Regel, es wird nichts gegessen und nichts getrunken.“ [L107TR1, Pos. 37])<br><br>**Hinweis:** Auch wenn die konzeptionelle Korrektheit teilweise für andere Indikatoren als Begründung fungiert, wird sie wie üblich kodiert.<br><br>**Hinweis**: Die Korrektheit/Inkorrektheit der LK-Aussage wird nicht berücksichtigt. D. h., wenn die LK fachlich falsche/inadäquate Aussagen macht, sich jedoch auf die konzeptionelle Korrektheit bezieht, wird dies kodiert. Bsp. „Ich konnte aufgrund

[1] Adamina, Marco; Kübler, Markus; Kalcsics, Katharina; Bietenhard, Sophia; Engeli, Eva (2018): Vorstellungen von Schülerinnen und Schülern zu Themen des Sachunterrichts und des Fachbereichs Natur, Mensch, Gesellschaft – Einführung. In: Marco Adamina, Markus Kübler, Katharina Kalcsics, Sophia Bietenhard und Eva Engeli (Hg.): „Wie ich mir das denke und vorstelle…“. Vorstellungen von Schülerinnen und Schülern zu Lerngegenständen des Sachunterrichts und des Fachbereichs Natur, Mensch, Gesellschaft. Bad Heilbrunn: Verlag Julius Klinkhardt, S. 7–20. Online verfügbar unter https://www.klinkhardt.de/newsite/media/20180821_9783781522572%20Adamina%20ua.pdf, zuletzt geprüft am 21.07.2022.

Zeitmangels das Protokoll nicht behandeln. Das ist für ein Experiment auf dieser Stufe meines Erachtens auch nicht sehr wichtig.“ [L108AR1:7]

Beispiele für Konzepte und Strategien: Einhaltung des Experimentier-Zyklus (Teilprozessansatz); Korrektheit des Problemtypens; Missachtung des VKS-Konzepts; Vorhandensein einer Messwiederholung; etc.

Fachliche Klärung
Sachgerechte Sprache

Die LK nimmt Bezug auf die korrekte/inkorrekte Verwendung oder Schwierigkeiten bei der Verwendung der Sprache bzgl. der Zielkonzepte zum ExpH (Fachsprache, Alltagssprache) bei sich oder den SuS.

Hinweis: Sprachliche Schwierigkeiten, z. B. durch unbekannte (Fach-)Begriffe werden nur dann kodiert, wenn klarer Bezug zu ExpH vorhanden ist (z. B. „Begriff Messwiederholung unbekannt“).

Hinweis: Wird der Bezug zu ExpH nicht eindeutig wird 77 kodiert. Dazu gehören bspw. Schwierigkeiten Ideen zu versprachlichen/festzuhalten (Hier wäre Idee vorhanden, aber kann nicht kommuniziert werden).

Hinweis: Die Korrektheit/Inkorrektheit der LK-Aussage wird nicht berücksichtigt. D. h., wenn die LK fachlich falsche/inadäquate Aussagen macht, sich jedoch auf die sachgerechte Sprache bezieht, wird dies kodiert.

Hinweis: Wenn LK lediglich auf kognitive Hürde der Sprache eingeht, dann nicht als sachgerechte Sprache kodieren, sondern als 77_kognitiver Anspruch (z. B. Lesen und verstehen: SuS haben Mühe mit Sprache, SuS hat LRS etc.).

Fachliche Klärung
Schüler:innen-gerechte Konzepte

Die LK nimmt Bezug auf die SuS-Gerechtheit der Konzepte zum ExpH. Die Konzepte müssen dabei auch fachlich korrekt elementarisiert sein oder es werden Unsicherheiten, Fehler erkannt und berichtigt. D. h. beispielsweise, dass VKS so erklärt wird, dass SuS diese verstehen bzw. erkannt wird, wenn es beim Verständnis Schwierigkeiten gab aufgrund der nicht-SuS-Gerechtheit. Die Aussagen können hierbei eine Diskussion der durchgeführten Aktivitäten in Bezug auf Verständlichkeit/Angepasstheit für die SuS oder eine Diskussion der zukünftigen Anpassung der Aktivitäten in Bezug auf bessere Verständlichkeit/Angepasstheit für die SuS beinhalten.
Hinweis: Es muss klar werden, dass die LK die Inhalte bewusst vereinfacht, anpasst bzw. herunterbricht (Intention der Aussage muss sein, Verständlichkeit/Angepasstheit für SuS zu diskutieren/erhöhen/sicherstellen). Dies soll nicht „per Zufall“ geschehen. (z. B. VKS wird bewusst vereinfacht, indem sie bildlich dargestellt wird oder an einem Beispiel durchgespielt wird.)

Hinweis: Nur zielgerichtetes Nachfragen reicht nicht aus, wenn nicht explizit heruntergebrochen wird. (z. B. nicht SuS-gerecht, sondern Umgang mit SuS-Vorstellungen):

〉 „Wie kannst du darauf schließen, dass sich die Asseln im Trockenen nicht wohlfühlen, nur weil sie die ganze Zeit herumrennen? Durch solche Fragen konnte ich sie darauf bringen.“ [Z314AR2:17, mod.]

Hinweis: Direktive Vorgaben/Lenkung werden nicht als SuS-gerecht kodiert. Bspw. wenn LK vorwegnimmt, was gemacht werden muss, ohne verständlich herunterzubrechen:

〉 „Bei zwei Gruppen musste ich klar sagen, trennt ab – einmal feucht, einmal trocken, dass im gleichen Raum beide Bedingungen vorhanden sind, weil sie nicht daraufgekommen sind.“ [Z314AR2:17, mod.]

Hinweis: ExpH-Konzepte = VKS, Messwiederholungen, Experimentierzyklus (Teilprozesse) etc.

Abgrenzung zu Kognitiver Anspruch:
- <u>SuS-gerecht</u> = wenn LK Bezug darauf nimmt, inwiefern <u>Konzept</u> heruntergebrochen wurde und dadurch verständlich ist/wird.
- <u>Kognitiver Anspruch</u> = Die <u>Aufgabe</u> ist durch die SuS lösbar (Zone der proximalen Entwicklung der SuS).

Unterrichtsziele
Zielauswahl

Die LK nimmt Bezug auf die Auswahl ihrer Lernziele (z. B. geeignet, ungeeignet, zufrieden, unzufrieden) oder nennt Gründe für ihre Auswahl. LK spricht über Anpassung/Änderung der Lernziele. Dazu gehört auch die Auswahl neuer Lernziele. Die LK spricht die Priorisierung ihrer Lernziele an („Es war mir wichtiger, dass Sie ...“ [L104TR1:31]).

Der Bezug kann auch eher indirekt geschehen, solange er klar ist (bspw. „[…] dass man sehr offen bleibt, bedingt eben auch, dass man nicht sehr stark auf Erklärungen eingeht und weil es so viele Möglichkeiten und Faktoren gibt, **geht es hier wirklich nur um den Prozess des Experimentierens**.“ [L101TR1: 33, angepasst]).

Hinweis: Werden die Lernziele lediglich eindeutig erwähnt oder wiederholt, jedoch ohne Bewertung, Begründung etc., wird nur Beschreibung kodiert. [Z205AR1:19]

Hinweis: Es sind ausschließlich die Lernziele gemeint, welche die Studierenden selbst gesetzt haben.

Hinweis: Wenn auf Lernziele referenziert wird, aber diese nicht explizit genannt werden, kann in der Planung nachgeschaut werden.

Unterrichtsziele
Zielorientierung

Die von der LK gestalteten Interventionen/der Lernweg zielen auf das ExpH (Zielkonzept) hin.

- LK nimmt Bezug darauf, inwiefern der Unterricht als Ganzes oder bestimmte Aufgaben dem Lernziel/den Lernzielen förderlich oder lückenhaft waren, bzw. die SuS zur Erreichung der Lernziele hingelenkt werden.
- LK beschreibt, inwiefern Sie die SuS beim Abdriften zurück auf die Lernziele gesteuert hat mit ihren Interventionen.

Hinweis: Die Zielorientierung muss zwingend eine **Intervention oder hypothetische Intervention** der Lehrperson umfassen. (Bspw.: „[...] Aber wie gesagt es ist schwierig, in einer vorbedachten Planarbeit zu bleiben und sich zu überlegen, wo setze ich die Grenze und sage hier ist jetzt wirklich fertig, unsere Experimente haben doch auch Grenzen." [L101TR1: 5, angepasst]; „Die Experimente bringen eine gewisse Eigendynamik mit sich und diese Eigendynamik ähm bringt viele bereichernde Momente, aber auch Momente, wo es in eine Richtung läuft, die man entweder nicht geplant hat oder nicht möchte und es ist schwierig, diese Dynamik umzulenken in die Richtung, in die man möchte, ohne das autoritär vorzugeben." [L101TR1: 3, angepasst])

Abgrenzung zur Zielklarheit: Zielorientierung bezieht sich auf die Handlungen der LK. Es wird dabei nicht zwingend klar, ob für die SuS das Ziel klar ist. Die Intention der LK ist es, die SuS mit ihren Interventionen in Richtung Lernziel(e) zu lenken.

Hinweis: Muss nicht nur zwingend bewusste/geplante Handlung sein (nicht nur abdriften), sondern kann auch sein, dass LK darüber spricht, inwiefern „zufällige" Situationen im Unterricht erkannt (oder aufgegriffen) wurden die zu Lernzielen passen.

Hinweis: Faustregel: Wenn Frage „Ist es förderlich für Lernziel-Erreichung" beantwortet wird, ist es i. d. R. Zielorientierung.

Hinweis: Die Zielorientierung muss in Bezug auf die gesetzten Lernziele sein. Wird die Zielorientierung in Bezug auf neue Aspekte/Lernziele angesprochen, wird nicht Zielorientierung kodiert. **Ausnahme:** Wird die übergeordnete Projekt-Kompetenz zu Experimentieren angesprochen, dann werden die Aussagen unter Zielorientierung kodiert.

Hinweis: Es sind ausschließlich die Lernziele gemeint, welche die Studierenden selbst gesetzt haben.

Hinweis: Wenn auf Lernziele referenziert wird, aber diese nicht explizit genannt werden, kann in der Planung nachgeschaut werden.

Unterrichtsziele
Zielklarheit

Die LK äußert sich dazu, inwiefern sie der Meinung ist, dass den SuS die Lernziele verständlich waren oder nicht verständlich waren (bspw. SuS wussten (nicht), was Lernziele der Lektionen sind), bzw. dass klar wurde, was gemacht werden muss in Bezug auf das Lernziel oder warum eine bestimmte Aufgabe/Handlung gemacht wird (Zusammenhang mit Lernziel wird klar).

Hinweis: Wird angesprochen, dass Lernziele nicht kommuniziert wurden (kann auch 77 sein), dann ist es i. d. R. Zielklarheit.

Hinweis: Es sind ausschließlich die Lernziele gemeint, welche die Studierenden selbst gesetzt haben.

Hinweis: Wenn auf Lernziele referenziert wird, aber diese nicht explizit genannt werden, kann in der Planung nachgeschaut werden.

Unterrichtsziele
Zielerreichung

Die LK nimmt Bezug auf die Erreichung der Lernziele oder den Lernzuwachs. Dies können Aussagen dazu sein, ob (bestimmte) Lernziele erreicht wurden und ob sie zufrieden/unzufrieden damit ist (es muss klar sein, dass Lernziel ExpH betrifft). Die LK schätzt die Machbarkeit der Erreichung des Lernziels ein.
Sie macht Aussagen zur Überprüfung der Lernziele, z. B. Selbsteinschätzung/Selbstreflexion durch SuS, Überprüfung durch LK etc. (sofern klar wird, dass es Lernziele zum ExpH sind).

Hinweis: Es sind ausschließlich die Lernziele gemeint, welche die Studierenden selbst gesetzt haben.

Hinweis: Wenn auf Lernziele referenziert wird, aber diese nicht explizit genannt werden, kann in der Planung nachgeschaut werden.

Reflexivität
Prozessreflexion

Die LK beschreibt, inwiefern sie ein nachgelagertes Nachdenken über den Experimentierprozess bei den SuS festgestellt oder angeregt hat oder dies versäumt hat.
Zum Experimentierprozess gehören bspw. Eignung der Fragestellung, Relevanz der Forschungsfrage oder Planung, Berücksichtigung der Messwiederholung, VKS, Genauigkeit der Messungen, über den naturwissenschaftlichen Experimentierprozess nachdenken, was gehört zum naturwissenschaftlichen Experimentieren, was ist nicht naturwissenschaftliches Experimentieren etc.

Hinweis: Das Nachdenken über den Prozess muss nach der Umsetzung des Prozesses stattfinden. Wird währenddessen reflektiert (z. B. in korrekte Richtung lenken, SuS-Vorstellungen eruieren etc.) werden nur die entsprechenden Items kodiert.

Hinweis: Da nicht eindeutig festgestellt werden kann, ob SuS tatsächlich angeregt werden/mitdenken, reicht auch bereits die Intention der LK zur Anregung der SuS (bspw. mittels direktiven Inputs) aus.

- „Dann gegen Schluss (…) bin ich noch zum Teil drauf eingegangen auf gewisse Dinge, die man beim Experimentieren eben beachten sollte, wie dass man es nicht nur einmal durchführen sollte, sondern mehrmals, oder auch sonst, ob Schwierigkeiten oder Probleme aufgetaucht sind.“ [L201TR1: 13]

Hinweis: Wenn die SuS im Experimentierplanungsprozess notwendigerweise über Vorgehensweise nachdenken, zählt dies nicht als Prozessreflexion.

Hinweis: Prozessreflexion kann in jeder Phase des Unterrichts, geplant/spontan oder im Plenum/in Gruppen/einzeln stattfinden.

Hinweis: Abgrenzung zu häufigen „Doppelkodierungen“ mit Umgang mit SuS-Vorstellungen, konzeptionelle Korrektheit etc.: Reflexion sollte bewusst gewollt und einigermaßen strukturiert sein. D. h. nur mit Einzelfragen SuS auf Nachdenken über Prozess anzuregen wird nicht kodiert:

- „Ich hatte ja teilweise nochmal ein Gespräch, warum darf man jetzt nicht umrühren und zerkleinern, dass man immer wirklich nur eine Variable verändert und dass die restlichen Bedingungen gleich sein müssen. Das ist nicht so intuitiv, habe ich das Gefühl.“ (N202TR1: 15)
  → Lenkt SuS in korrekte Richtung bzgl. SuS-Vorstellung, denkt aber nicht über Prozess als solches nach
- „Und eben auch das mit einer Variable, die man nur verändern kann. Das musste ich bei einigen nochmal korrigieren, hatte ich das Gefühl. Wie ich damit umgegangen bin? Ich habe halt nochmal versucht zu erklären oder zu sagen, bist du dir jetzt sicher, wenn du hier jetzt umrührst und zerkleinerst, dass es wegen dem Zerkleinern kommt? Oder könnte es auch jetzt wegen dem Umrühren kommen? Woran kannst du das jetzt festmachen, was jetzt das schnellere Auflösen ausgelöst hat? (…) Also so mit Rückfragen, dass sie draufkommen, hoffentlich.“ (N202TR1, Pos: 17)

Hinweis: Wird fehlende Reflexion angesprochen und es wird klar, dass es eine Prozessreflexion betrifft, so wird mit Indikator kodiert.

Hinweis: Zum Teil wird hier sehr generisch von „Nachdenken über das Experimentieren/Weiterdenken“ gesprochen ohne weitere Details. Es muss einerseits klar werden, dass es nicht um die Ergebnisse geht, sondern um den Prozess. Und andererseits, da die Interviewfrage teils triggert, reicht es nicht aus, generisch und ohne Details darüber zu sprechen. Wird nicht eindeutig klar, dass es eine Prozessreflexion ist, wird mit „Ergebnisreflexion“ kodiert. Wird zusätzlich der Bezug zu ExpH nicht eindeutig gemacht, wird mit „77_Ergebnisreflexion“ kodiert (z. B. fachwissenschaftlicher Fokus oder nur allg. über Ergebnisse sprechen) („Ich hatte keine Zeit mehr für die Reflexion“ [Z304TR1: 7]).

Reflexivität

Ergebnisreflexion

Die LK beschreibt und erläutert Möglichkeiten für ein Nachdenken über die Ergebnisse und bespricht diese bezüglich des Zielkonzepts (ExpH). Unterschiedliche Ergebnisse werden verglichen, berücksichtigt und diskutiert.

Bspw. stellt LK die Resultate der Gruppen gegenüber, mit der Absicht (geplant oder spontan) zu zeigen, warum z. B. Messgenauigkeit, Messwiederholung etc. relevant sind. → Ergebnisse der Aufgaben werden mit Methode des Experimentierens in Bezug gesetzt.

Hinweis:

- ExpH → Nachdenken über die Ergebnisse im ExpH-Sinn (fachmethodisch); Ergebnisreflexion geschieht in Bezug auf ExpH-Konzepte. In Ordnung, wenn LK Resultate als Beantwortung der Forschungsfrage verwendet. Es muss aber explizit klar gemacht werden, dass dies dem Experimentierablauf dienen soll (bspw. Zweck von Experimenten zur Beantwortung einer Forschungsfrage).
- 77 → fachwissenschaftliche Reflexion zu Assel- oder Tabletten-Zusammenhängen anhand der Ergebnisse (fachkontextorientiert/fachwissenschaftlich orientiert). Bspw. Hauptinhalt, dass SuS nun wissen, wo Asseln gerne leben oder Brausetabletten sich am schnellsten lösen.

Hinweis: Dies muss nicht nur am Schluss im Plenum geschehen, sondern kann in jeder Phase des Unterrichts, bspw. spontan in Gruppen, geschehen.

Hinweis: Doppelkodierungen mit Prozessreflexion sind möglich (z. B. wenn Nachdenken über unterschiedliche Resultate zu einem Nachdenken über Experimentierprozess führt).

Hinweis: Es muss eindeutig klar sein, dass es eine Ergebnisreflexion ist. Wenn z. B. nur allg. das Sprechen über Ergebnisse angesprochen wird, reicht dies nicht aus (bspw. „Am Schluss haben wir noch über die Ergebnisse gesprochen.“) → kodieren mit „77_Ergebnisreflexion“

Reflexivität

Begriffsbildung

Die LK thematisiert die Begriffsbildung/Begriffsbedeutung in Bezug auf die Zielkonzepte des ExpH (Klärung der Bedeutung von relevanten Begriffen, Bedeutungsunterschiede von Begriffen in der Alltags- und Fachsprache, Korrekte Verwendung/adäquate Ausdrucksweise, Herkunft der Begriffe etc.).

Hinweis: Die Begriffsbildung muss nicht zwingend in Fachsprache geschehen, sondern kann auch in Alltagssprache passieren.

Abgrenzung zu „sachgerechte Sprache“: Nicht kodiert wird, wenn es nur um die Bekanntheit des Fachwortschatzes geht (z. B. Begriff „Petrischale“ kennen). In diesem Fall wird „sachgerechte Sprache“ vergeben. Bspw. wenn die LK eine Begriffsliste mit Erklärungen (Bedeutungen) abgibt, wäre das Begriffsbildung. Gibt sie hingegen nur eine Wörterliste ab (Fachwortschatz) ist dies „sachgerechte Sprache“. → Es muss um das Verständnis von dahinterliegenden Konzepten gehen (z. B. Konzept des naturwissenschaftlichen Experimentierens, VKS etc.).

Didaktische Strukturierung<br>**Emotionale Situierung**
**Die LK thematisiert, inwiefern Sie mit Hilfe von Kontextualisierung/Aufgaben (vgl. L105TR2: 3) (z. B. Hinweise zur Relevanz, Brauchbarkeit, zum Alltagsbezug) emotionale Reaktionen der SuS für das Zielkonzept ExpH auszulösen versucht hat, dies geschafft hat oder dies unterließ. Die LK spricht hierbei an, inwiefern für die SuS die Wichtigkeit/Zweck des ExpH für die SuS selbst klar wurde oder wo sie Motivation/Interesse/Desinteresse festgestellt hat.**<br><br>**Hinweis:** Allgemeine Aussagen (z. B. „Die SuS waren motiviert") werden kodiert, sofern der Bezug zur emot. Sit. klar wird: Kodierung mit 77.<br><br>**Hinweis:** In Ordnung, wenn LK auf Fachkontext (A oder T) referenziert, solange die Aussage sich auf ExpH bezieht (bspw. „Also nicht, weil sie nicht arbeiten wollten, sondern weil sie sich geekelt haben. Darum habe ich es für sinnvoll erachtet, ein bisschen größere Gruppen von drei bis vier zu machen, damit wenigstens ein, zwei Personen pro Gruppe sich wirklich auch trauen, etwas zu machen." [L106AR1: 11] → ExpH-Bezug muss klar sein (z. B. aus Kontext))<br><br>**Hinweis:** Spricht die LK nicht die emotionale Situierung <u>der SuS</u> an, sondern nur die eigene, wird nicht mit emotionaler Situierung kodiert. Bspw. „Ich bin zufrieden. Das Experimentieren mit der Klasse hat mir sehr Spaß gemacht." [N208AR1:5, mod.]<br><br>**Hinweis:** Wenn genannter Ekel (erfolgreiches) Experimentieren nicht beeinflusst, dann wird 77 kodiert.

Didaktische Strukturierung<br>**Kognitiver Anspruch**
**Die LK macht Aussagen dazu, inwiefern die gestalteten Interventionen und Aufträge aus SuS-Perspektive angemessen waren oder wo diese über- bzw. unterfordert oder herausfordernd waren (bspw. Offenheit). Sie nimmt Bezug auf Schwierigkeiten im Aufgabenmaterial (z. B. bei Arbeitsblatt) oder eine herausfordernde und interessante Gestaltung für die SuS etc. Der Anspruch kann bspw. durch mangelndes Vorwissen, Rahmenbedingungen, immanente Schwierigkeiten etc. diskutiert werden.**<br><br>**Hinweis:** Ist die Aufgabe/der Unterricht für die SuS lösbar/bewältigbar, meint jedoch nicht Klarheit der Instruktion oder Zielklarheit<br><br>**Hinweis:** Wird allgemein von Über-/Unterforderung gesprochen, ohne dass ein Bezug vorhanden ist, wird nicht kodiert: „Eigentlich nicht groß differenziert" → <u>keine Kodierung</u>, weil zu allgemein; Differenzierung angesprochen und (leicht) begründet → <u>Indikatoren vergeben (Falls kein ExpH-Bezug → 77)</u><br><br>**Beispiel:** Der kognitive Anspruch muss in Bezug auf ExpH sein. Bspw. nicht-ExpH = wenn sprachliche Überforderung aufgrund von LRS. Wenn es aber darum geht, dass kognitiver Anspruch zu hoch ist, weil unbekannte Begriffe zu ExpH nicht eingeführt, Aufgabe unklar etc. dann ist es ExpH vgl.:<br>〉 „Überforderungen gab es ab und zu mal, genau bei diesem Arbeitsblatt mit den Forschungsaufträgen oder auch beim völlig offenen Experimentieren. Zum Beispiel: Was sollen wir machen? oder halt auch die Wörter nicht verstanden, Aufgaben nicht verstanden." [Z201AR1:27, mod.]<br><br>**Hinweis:** Wird explizit erwähnt, dass SuS mehr aktiviert werden sollen, wird kognitiver Anspruch vergeben (Bezug ExpH klar → Indikatoren; Bezug ExpH unklar → 77)<br><br>**Abgrenzung zu SuS-gerecht: SuS-gerecht** = Wenn LK Bezug darauf nimmt, inwiefern <u>Konzept</u> heruntergebrochen wurde und dadurch verständlich ist/wird. **Kognitiver Anspruch** = Die <u>Aufgabe</u> ist durch die SuS lösbar (Zone der proximalen Entwicklung der SuS).<br>〉 „Ich habe da noch einen Text erstellt, warum sich die Brausetablette in warmem Wasser schneller löst als in kaltem und bin da auf die Aggregatszustände eingegangen. Sie kennen aber nur das Teilchenmodell." [Z1314TR1:7, mod.]<br><br>**Abgrenzung zu sachgerechte Sprache:** Wenn LK lediglich auf kognitive Hürden der Sprache eingeht, dann nicht als sachgerechte Sprache kodieren, sondern als 77_kognitiver Anspruch (z. B. Lesen und Verstehen: SuS haben Mühe mit Sprache, SuS hat LRS etc.).

Didaktische Strukturierung<br>**Sequenzierung und Strukturierung des Lernprozesses**
**Die LK nimmt Bezug darauf, inwiefern sie den Lernprozess zum ExpH als in Bezug auf Sachlogik/Aufbau/Reihenfolge sinnvoll/unsinnig strukturiert/sequenziert (=Aufteilung des Lerninhalts in sinnige und aufbauende Schritte) wahrnimmt (dies kann bspw. anhand Tiefen-Struktur-Map, LUKAS, Experimentierzyklus, Problemtypen geschehen). Die Elementaren Grundideen werden nachvollziehbar verknüpft und sinnvoll in vorherige und nachfolgende Lektionen eingebettet (Fachimmanente Einbettung), D. h. auch inhaltlich sinnvolle Überleitungen.**<br><br>Zu diesem Indikator gehört auch, wenn die LK diskutiert, dass gewisse Aspekte (bspw. Wissensvoraussetzungen, technische Fähigkeiten, Zeitressource, Nachvollziehbarkeit/Lückenhaftigkeit bei Aufbau von Inhalten/Aufgaben) im Lernprozess mangelhaft/fehlend waren und dadurch Auswirkungen auf weitere Teile des Lernprozesses hatten.<br><br>**Hinweis:** Wenn LK Zeitpunkt der Materialeinführung mit klarem Bezug zu Sequenzierung/Strukturierung und ExpH anspricht, wird mit Material parallelkodiert.<br>〉 „Ich hätte vor Erarbeitung der Fragestellung <u>zuerst das Material verteilen</u> sollen damit sie sehen, <u>welches Material steht mir zur Verfügung</u>." [Z204AR2:9, mod.] → Spricht sinnvollen Ablauf an, um Voraussetzungen zu gewährleisten

Hinweis: Wenn Auswirkungen der Zeit auf die Sequenzierung/Struktur angesprochen wird, werden „Zeit am Auftrag“ und „Seq./Strukt.“ doppelkodiert.

Hinweis: Wird die Struktur lediglich als Aspekt der Zeitallokation angesprochen, jedoch wird die Struktur nicht angepasst/diskutiert, dann wird nur „Zeit am Auftrag“ kodiert.

Hinweis: Ganz allgemeine Aussagen in Bezug auf die Struktur werden nicht kodiert, da Indikatorzugehörigkeit nicht eindeutig geklärt werden kann (z. B. „Ich würde nichts an meiner Planung ändern“).

Didaktische Strukturierung
Zeit am Auftrag

Die LK nimmt Bezug auf die aktive Zeit (time on task), welche die SuS für das ExpH zur Verfügung hatten (umfasst z. B. auch Planungsphasen aus dem Experimentierzyklus) oder sie spricht die Anpassung der Zeitallokation in Bezug auf den Experimentierprozess an (bspw. „mehr Zeit für Durchführungsphase notwendig, dafür etwas weniger für Planungsphase“ → Aufbau bleibt gleich, zeitliche Allokation wird verändert).

Hinweis: Wenn Auswirkungen der Zeit auf die Sequenzierung/Struktur angesprochen wird, werden „Zeit am Auftrag“ und „Seq./Strukt.“ doppelkodiert.

Hinweis: Wird die Zeitallokation im Zusammenhang mit der Sequenzierung/Struktur angesprochen, die Struktur wird jedoch nicht angepasst/diskutiert, dann wird nur „Zeit am Auftrag“ kodiert.

Hinweis: Ganz allgemeine Aussagen in Bezug auf die Zeit werden nicht kodiert (z. B. „Ich habe den Zeitplan (nicht) eingehalten“ (vgl. [Z204AR2:5, mod.]), „Das Zeitmanagement hat nicht funktioniert“).

Didaktische Strukturierung
Material

Die LK nimmt Bezug darauf, inwiefern sie die Einführung/Handhabung/Auswahl des Materials von sich selbst oder den SuS als passend/unpassend empfunden hat. Sie nimmt Bezug darauf, wie sie eine korrekte Verwendung des Materials sichergestellt hat (z. B. Regeln für den Umgang mit den Asseln), eine inkorrekte Verwendung bei sich oder den SuS festgestellt hat oder was sie diesbezüglich anpassen würde. Sie nimmt Bezug darauf, inwiefern sie oder die SuS das Material zielführend und korrekt eingesetzt haben. Erkannte Schwierigkeiten in der Handhabung oder in der Auswahl werden angesprochen.

Hinweis: Die Asseln zählen vereinfachend zum Experimentiermaterial, wohlwissend um deren lebenden Charakter.

Hinweis: Die Aussagen zum Material sollen einen Bezug zum ExpH aufweisen und nicht nur dem Fachkontext dienlich sein:
- ExpH = wenn Materialauswahl/-ergänzung etc. dem Experimentieren dienlich ist oder beeinflusst
- Nicht-ExpH = wenn Materialzusatz lediglich weitere Variablen abdeckt

Hinweis: Wenn Ablenkung durch Material angesprochen wird, wird mit „Material“ kodiert.
〉„Ich hätte die Asseln später ausgeteilt, weil die einen haben einfach ausprobiert.“ [Z306AR2:33, mod.] → ExpH, weil „einfach ausprobiert“ auf mangelnde Einhaltung Experimentierprozess hinweist.

Hinweis: Wenn LK Zeitpunkt der Materialeinführung mit klarem Bezug zu Sequenzierung/Strukturierung und ExpH anspricht, wird mit Sequenzierung/Strukturierung parallelkodiert.

Hinweis: Wenn LK lediglich erwähnt, dass keine Änderungen beim Material „Ich würde nichts ändern.“ → nichts kodieren;
Wenn LK beschreibt/begründet warum keine Änderungen → 77

Hinweis: Wenn zusätzliche Schwierigkeiten/Vorteile des biologischen/chemischen Kontextes in Bezug auf das Experimentieren diskutiert werden (kann sich auf SuS oder LK beziehen), wird mit „Material“ kodiert.
〉„Das Experiment ist schwieriger zum Durchführen, da die Asseln willkürlich reagieren und die Aussagen unterschiedlich ausfallen.“ [Z205AR1:41, mod.]; „Es war für mich schwierig, da ich selber nicht wusste, wie ich mit den Asseln umgehen soll.“ [fiktiv]
